# Probing the habitability of potential sulfuric acid rich subsurface lakes in Europa's ice shell via *Saci*/STIV integrated models

**DOI:** 10.3389/fmicb.2026.1849852

**Published:** 2026-05-29

**Authors:** Damara Saggio, Christine L. Phung, Moisés Bravo, Timothy C. Corcoran, Jamie C. Snyder, S. Chantal E. Stieber

**Affiliations:** 1 Department of Biological Sciences, California State Polytechnic University, Pomona, CA, United States; 2 Department of Chemistry & Biochemistry, California State Polytechnic University, Pomona, CA, United States

**Keywords:** astrobiology, biosulfur, chemolithotrophy, Europa, ice shell, STIV, Sulfolobales

## Abstract

Experimentally modeling the habitability of extreme ocean world conditions, such as those on Jupiter's moon: Europa, would benefit from integrated microbe-virus model studies to provide both a microbial model and a virus capable of nutrient cycling. This work experimentally probes the microbe/virus combination *Saci*/STIV as a potential microbial model for habitability studies of Europa's ice-ocean interface in subsurface lakes with implications for habitats with extreme conditions and fluid mixing. This study experimentally modeled archaea/virus-integrated systems in one set of conditions that fall within the range of currently proposed for subsurface lakes in Europa's ice shell: 3.5% salinity, pH 3, rich in Na^+^/Fe^2+^/Mg^2+^/${\text{SO}}_{4}^{2-}$/H^+^, fluctuating dissolved oxygen (D.O.), and 1–2 °C to explore habitability limits and candidate biosignatures. Planktonic *Sulfolobus acidocaldarius (Saci*) lysogens of *Sulfolobus* Turreted Icosahedral Virus (STIV) were included for the determination of potential habitability and biosignatures in both models. Extensive biofilm formation, biosulfur detection via Raman spectroscopy, viral mRNA/protein detection by RT-qPCR and western blotting, and successful viability assays under these conditions support the potential habitability under Europa-analog conditions. Cold-adapted *Saci* developed extensive biofilm rich in biosulfur globules, suggesting the possibility of sulfur-oxidizing metabolism. Finally, biosulfur and viral major capsid proteins (MCPs), with inter-domain conserved double jelly roll morphologies, were identified as candidate biosignatures for life on acidic icy ocean worlds. Such cryotolerant strains and the viral proteins proliferated from them may have applications in astrobiology, but also for extreme microbiology and beyond.

## Introduction

Determining if microbial life is harbored in other ocean worlds is experimentally challenging, often limited by the lack of reference data both from the target body and from experiments on Earth, but has implications for understanding life in extreme conditions. Sea ice ecosystems, including ice ocean interfaces and ice shell subsurface lakes, are possible habitable environments both on Earth and in other ocean worlds that have extreme conditions and fluid mixing. Experimental models of such systems could inform our understanding of microbial life on Earth, as well as generate potential biosignatures for astrobiology with insights into the habitability of other ocean worlds. The work presented herein was modeled using conditions that fall within the range of conditions proposed for Europa's ice shell subsurface lakes based on current knowledge, however the models have broad applicability for probing habitability in other systems with harsh conditions.

### Ice shell habitability

**Figure 1:**
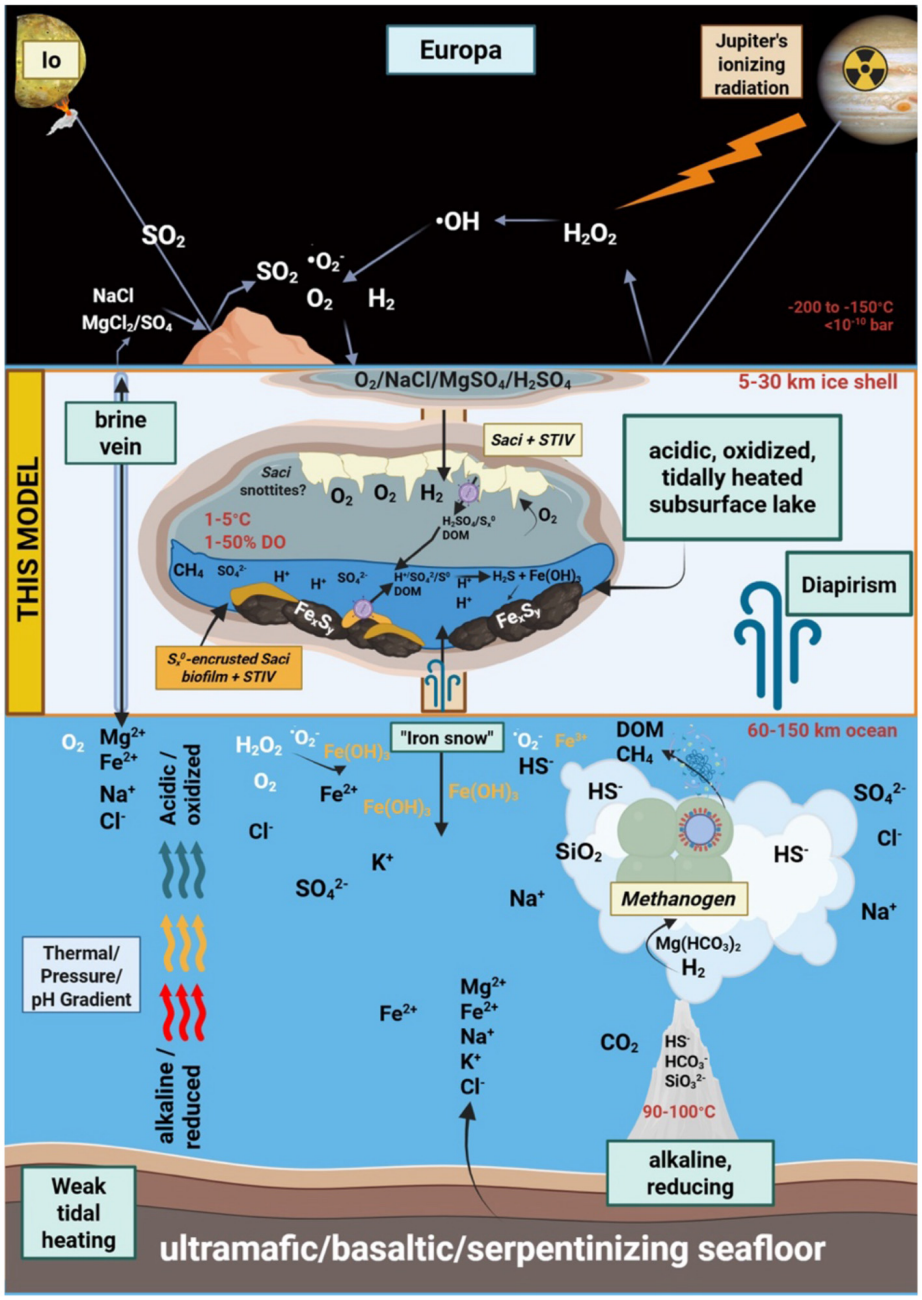
Potential Habitability Model of Europa's ocean and ice shell.

Europa is one of the few satellites in our solar system that is proposed to harbor all three key ingredients for life: water (Roth et al., 2014; National Academies of Sciences, and Medicine, 2023), energy (Roth et al., 2014; Russell et al., 2017), and redox chemistry (Russell et al., 2014, 2017; Vu et al., 2020; Sahai et al., 2024). *Galileo* flyby data suggests Mg-Na-Cl ocean emissions based on near infrared mapping spectrometry (NIMS) measurements (Spaun and Head III, 2001; Dalton et al., 2003), and an iron-rich core based on density measurements (Carr et al., 1998; Spaun and Head III, 2001; Vu et al., 2020; Thompson et al., 2021; Sahai et al., 2024). Bioavailable sulfur species are expected and are likely derived from volcanic SO_2_ emissions from Io, another of Jupiter's four moons (Figure 1). *Hubble* spectrograph data (Hall et al., 1995; Lewis et al., 2021) further proposes an oxidant rich exosphere consisting of radical oxides, and molecular oxygen via photo/radiolysis caused by Jupiter's powerful ionizing radiation (Hand and Brown, 2013). Finally, Europa's young/smooth surface suggests internal conductivity, and thus: a likely exchange of materials between its exterior and interior via tidal heating. Thermal gradients within Europa's ice shell have been computationally modeled (Zolotov and Shock, 2004; Gomez-Buckley et al., 2022; Vilella et al., 2020), promoting not just horizontal lenses and cryo-magma reservoirs, but also brine veins capable of salt and oxidant exchange between the interior and exterior (Pappalardo et al., 1998).

Europa's ice shell is predicted to be between 5 and 30 km thick (McKinnon, 1999), and may contain tidally heated subsurface lakes (Pappalardo et al., 1998; Manga and Michaut, 2017; Lesage et al., 2025; Walding et al., 2025). These putative subsurface lakes are predicted to be acidic and oxidized, harboring the necessary ingredients for lithotrophic life (Pappalardo et al., 1998; Manga and Michaut, 2017; Lesage et al., 2025; Walding et al., 2025). Recent studies of tidal friction and ice-shell kinetics predict that under certain conditions, subsurface lakes can reach temperatures of up to 1–5 °C (Chivers et al., 2021; Lawrence et al., 2024) similar to that of Earth's ocean. The diffusion of molecular oxygen and sulfur compounds from the surface ice to subsurface lakes via tidal fracturing within Europa's convective ice shell (Pappalardo et al., 1998; Walding et al., 2025) may support aerobic or microaerophilic microbes capable of sulfur redox metabolism (Figure 1). However, we are unaware of prior experimental laboratory attempts that assess potential chemolithotrophy within Europa's ice shell lakes.

Exogenous introduction of sulfur from Io onto Europa's surface could saturate small subsurface lakes as an energy source for chemolithotrophic life (Carr et al., 1998; Pappalardo et al., 1998; Vance et al., 2023). Iron oxyhydroxides or “iron snow” along the ice-ocean interface of Europa has also been proposed as an energy source for life (Figure 1), provided that dissolved ferrous iron from basaltic rock and ice fractures act as a highway for oxidants from the surface (Sahai et al., 2024). Considering that both carbon dioxide (Trumbo and Brown, 2023) and magnesium salts (Dalton et al., 2003; Brown and Hand, 2013) were detected on Europa's surface from *Keck* and *Galileo* observations, fluid flow from the ocean to the surface and vice versa becomes likely (Hand and Brown, 2013; Retherford et al., 2018; Trumbo and Brown, 2023). Consequently, these models and observations support possible Fe/S redox metabolism as a potential source of energy should life exist within Europa's ice shell.

### Previous ocean world models

**Figure 2:**
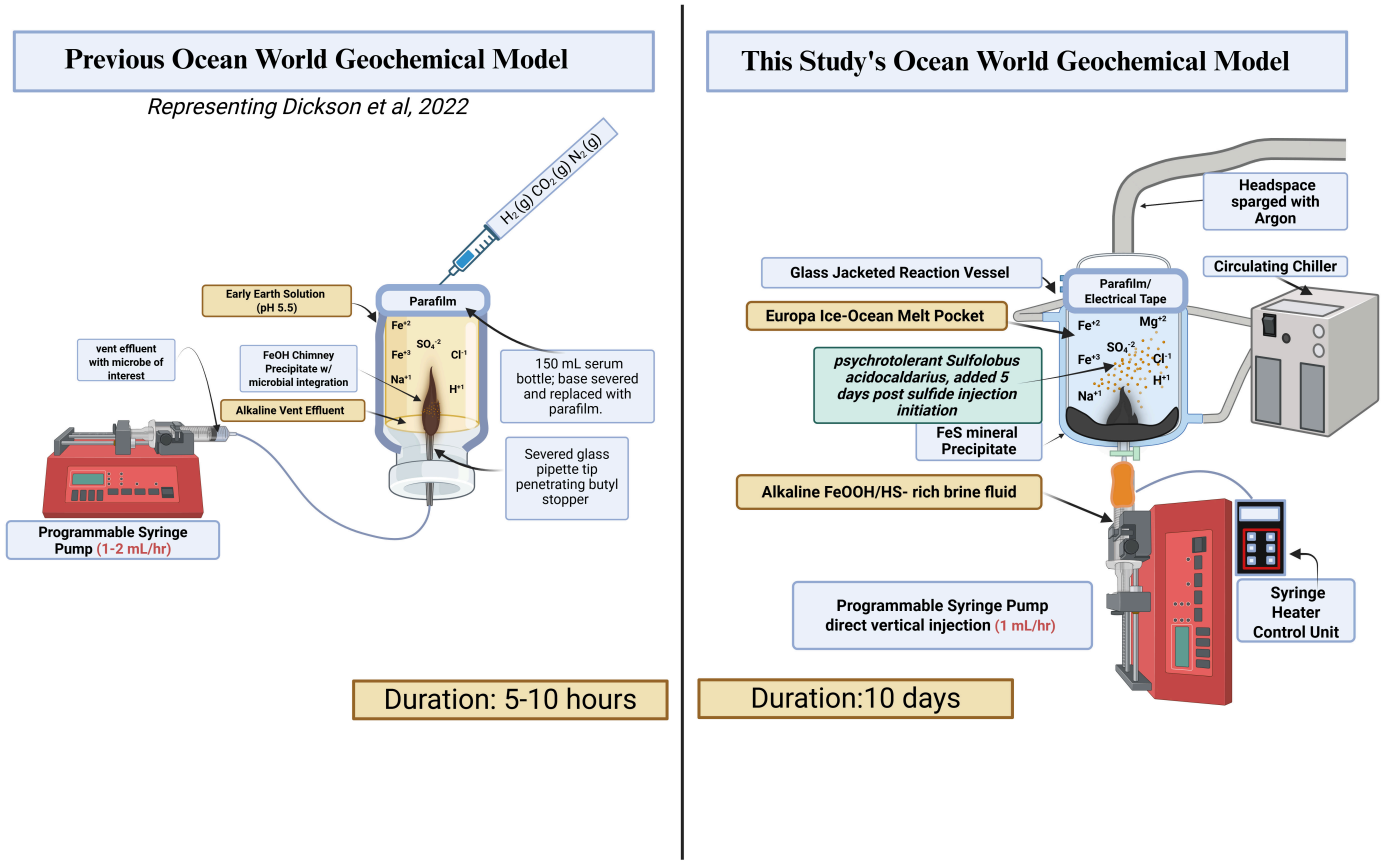
Previous vs. current microbe-integrated ocean world model setups. Previous models **(left)** include ambient temperatures, hydroxide effluent, circumneutral pH's, and effluent-bacteria integration (Barge et al., 2015; Hermis et al., 2021; Dickson et al., 2022). This study **(right)** includes thermal gradients, FeOOH/sulfide rich effluent (brine fluid), acidic reservoir pH, and planktonic lake extremophile integration.

Models of FeS-based geochemistry on Europa are considerably less studied than serpentinization chemistry, because Europa's cooler, ultramafic mantle suggests that hydrothermal energy is unlikely (Byrne et al., 2026). While most modern hypotheses favor a reducing/circumneutral ocean, similar to modern Earth, early *Galileo* hypotheses based on NIMS data promoted an acidic and oxidizing ocean (Dalton et al., 2003; Zolotov and Shock, 2004; Pasek and Greenberg, 2012). Similar to early Earth models, iron oxidation coupled to sulfate reduction have also been computationally modeled for Europa mineralization studies (Zolotov and Shock, 2003, 2004). Iron rich microbe-integrated vent models (Figure 2) have been reported primarily for early Earth studies, as opposed to Europa, and included bacterial injection of *Vibrio harveyi*, but did not assess long-term microbial viability beyond 10 h of incubation (Dickson et al., 2022). While these same FeS models have been proposed for Europa's ice-ocean interface (Russell et al., 2014, 2017) and seafloor (Barge and White, 2017; Hermis et al., 2021), the high predicted seafloor pressures of 110+ MPa for Europa's seafloor (Brown and Hand, 2013; Thompson et al., 2021; Hesse et al., 2022) suggest that Europa's convective ice at lower pressures could be more habitable for extreme microbes, and be possibly a more detectable zone for future landers and probes (McKinnon, 1999; Chivers et al., 2021; Lawrence et al., 2024). Previous microbe-integrated habitability models examined extremophiles in Europa's radiation laden surface (Dalton et al., 2003) and in more alkaline conditions (Taubner et al., 2018, 2020; Dickson et al., 2022), but to our knowledge, no studies have probed the habitability and biosignature potential of conditions that may describe Europa's subsurface lakes with archaea and their viral pathogens.

### A possible microbial model for Europa

Fe/S metabolizing microbial models found on Earth could help evaluate habitability of Europa-like conditions, and possible biotic vs. abiotic geochemical signatures (MacKenzie et al., 2020; Barge et al., 2022; Hand et al., 2022; Vance et al., 2023; Weber et al., 2023; Pappalardo et al., 2024). *Sulfolobus acidocaldarius* (*Saci)* are found in extreme environments such as acidic hot springs (Fernandes-Martins et al., 2024a,b), geyser basins (Sakai and Kurosawa, 2018), and sulfur rich fumeroles (Baquero et al., 2020). *Saci* thrive in acidic conditions (Rice et al., 2001; Dalton et al., 2003; Snyder et al., 2013), oxidize iron sulfides, tolerate both high and low D.O. conditions (Bischof et al., 2019), are capable of organo-heterotrophy and chemolithotrophy (Manga and Michaut, 2017; Tan et al., 2021; Logares, 2024; Lesage et al., 2025), and have metabolic oxidation capabilities with hydrogen sulfide gas (H_2_S), polysulfides (S_x_), elemental sulfur (S^0^), and sulfur minerals, under hypoxic and aerobic conditions (Sakai and Kurosawa, 2018; Liu et al., 2021; Fernandes-Martins et al., 2024a,b; Table 1). A previous study reported viability of *S. shibatae* at low temperatures (−80 °C), in nutrient dense Brock's media, and at pH 2.3, however survivability assays proved unreliable given the possibility of freeze-thaw during sample transport (Dalton et al., 2003). Finally, Sulfolobales are also easily cultivated in the laboratory, relative to their archaeal counterparts (McCollom, 1999; Harris and Schuerger, 2025), making them candidate microbial models for astrobiology.

**Table 1:** Sulfolobales tolerance zones.

Parameter	Current *Saci* tolerance zones	Proposed ice shell subsurface lake
Temperature	55–85 °C (Brock et al., 1972)	near 0 °C (Chivers et al., 2021; Pappalardo et al., 1998)
pH	1.5–5 pH (Brock et al., 1972)	2–6 pH (Dalton et al., 2003; Zolotov and Shock, 2004; Pasek and Greenberg, 2012)
Salinity	0–400 mM NaCl (Ye et al., 2020)	400+ mM NaCl (Brown and Hand, 2013)
D.O.	1.5–24% D.O. (Simon et al., 2009; Fernandes-Martins et al., 2024a)	Aerobic, microaerophilic (Pasek and Greenberg, 2012; Sahai et al., 2024)

### The need for a virus-integrated microbial model

Viral lysis of archaea is critical to maintaining biogeochemical cycles in Earth's oceans and is responsible for almost 1/3 of the oceans' recycled microbial biomass (Berliner et al., 2018; Lopez-Simon et al., 2023). Thus, virus-mediated biogeochemical cycling, or viral shunt, may be relevant for habitability in oceans on other worlds. Since Europa's ocean is likely twice the size of Earth's, consistent cycling of carbon, iron, and sulfur will be critical for supporting various marine zones (McCollom, 1999; Hand et al., 2007; Vance et al., 2016), and viral lysis may be a mechanism for this (Figure 1). One computational model of Europa's ocean floor included viral shunt for sustainable carbon cycling between the serpentinizing seafloor to a biofilm covered ice-ocean interface (Gomez-Buckley et al., 2022). While most archaeal viruses in the ocean are lytic and do not remain in the host for extended periods, temporal regulation of viral lysis and host cell organic matter (DOM) release may be beneficial in ocean systems on other worlds where conditions are more favorable in heavily populated microbial zones (Pappalardo et al., 1998; Chivers et al., 2021; Walding et al., 2025). As a model archaeal virus, STIV is well-studied (Rice et al., 2004; Snyder et al., 2011, 2013; Wirth et al., 2011; Fu and Johnson, 2012; Maaty et al., 2012), capable of withstanding extreme conditions (Snyder et al., 2011, 2013), and experimental propagation and purification methods are optimized (Snyder et al., 2011; Wirth et al., 2011; Maaty et al., 2012). While *S. solfataricus* is a commonly used *Sulfolobus* strain that is susceptible STIV infection (Redder and Garrett, 2006), *Saci* was chosen here due to a more stable genome, and as a reliable lysogen for STIV, allowing for temperate (lytic and lysogenic) virus life cycles necessary for temporal regulation (Anderson et al., 2017). For astro-virology work, STIV is a strong option because the *Saci* host is easy to cultivate, propagation via pH shock is standardized (Ortmann et al., 2008; Khayat et al., 2010), and downstream transcriptomic/proteomic analyses are established (Ortmann et al., 2008; Snyder et al., 2011; Maaty et al., 2012).

### This study

This work experimentally integrates an extremophile and a viral pathogen into aqueous models that include mixing fluids for a redox active interface. Parameters proposed for Europa subsurface lakes were applied to assess the microbial viability and habitability in a constrained set of possible conditions. While the current understanding of Europa's conditions has a large degree of uncertainty, this work presents a starting point to begin assessing habitability proposed for Europa. The *Saci/STIV* archaea/virus system was grown in a liquid vent system with parameters based on ranges of parameters proposed for Europa's acidic subsurface lakes within Europa's ice shell. Conditions included oxidants and sulfuric acid from the surface (Figure 1), as well as reducing ocean fluid with sulfides and “iron snow” vertically convected from the ice-ocean interface (Sahai et al., 2024). The presence vs. absence of added trace simple sugars were probed to understand the effects of carbohydrates on *Saci* viability and virus propagation. This work aimed to ascertain whether physicochemical stress and/or nutrient limitation were factors in microbial and viral viability under Europa analog conditions. To our knowledge, *Saci* and its virus, STIV, have not been previously studied for habitability experiments in harsh subsurface lake conditions. Developing experimental models with microbes in acidic ice-ocean world conditions would provide insight into habitability on Early Earth and other icy ocean worlds throughout our solar system. This is significant for assessing which biogeochemical/physicochemical dynamics could be promoting or preventing the viability of microbial life in extreme environments and other ocean worlds. Provided its hydrothermal model influences (Barge et al., 2015; White et al., 2015; Hermis et al., 2021; Dickson et al., 2022), this study presents additional methods of acidic ocean world studies in a hydrothermal and/or subsurface lake context.

## Materials and methods

### General considerations

The rationale for the selection of conditions and ranges is included in the Results section.

### Europa ice-ocean reservoir (lake) model setup

A 250 mL custom-made, glass-jacketed reaction vessel (Adams and Chittenden Scientific, Berkley, CA; UVM Glass Shop, Burlington, Vermont) was placed directly above a syringe pump, which was modified from previous experiments that used a horizontal syringe pump (Barge et al., 2015; Hermis et al., 2021; Dickson et al., 2022). A heated syringe that punctured an airtight rubber septum was vertically aligned to the vessel to prevent heat loss in effluent between systems (Figure 2). A D.O. probe (Vernier Optical, Beaverton, OR*)* was used to monitor D.O. % while sparging and before and after each trial. An effluent temperature of 80 °C was maintained at the syringe via a syringe heater control unit (New Era Pump Systems) to prevent effluent within the syringe needle from freezing, while a circulating chiller (Fisher Isotemp 3006) connected to each glass jacketed vessel was used to keep the lake simulant within the 1.2–1.7 °C throughout each 10-day trial.

Prior to adding the acidic lake fluid, the reaction vessels were rinsed three times with 10% HCl/MeOH solution. The vessels were then rinsed twice with ddH_2_O prior to acidic lake fluid (135 mL) introduction. Each chilled vessel was then purged with ultra-high purity (UHP) argon and sparged to a lake D.O. of 4.99% or less. D.O. saturations were ensured for each reaction vessel using a Vernier Optical D.O. Probe. Two layers of VQR Labeling Tape, followed by an additional two layers of Scotch electrical tape were applied to the top of the lake vessel (sealed by parafilm during sparging) to maintain a microaerophilic environment throughout the duration of the experiment. No further sparging was required to maintain microaerophilic (5–25% D.O.) conditions throughout the 10-day experiment, except during *Saci* inoculation, when UHP argon was held above the point of entry for 30 s or less. The D.O. slightly increases over the 10 days within the 5–25% D.O. range, with potential leakages through electrical heating tape and parafilm cap or via the plastic syringe. Continual sparging of UHP argon during the 10-day course of the experiment resulted in higher D.O. levels, so this approach was not used.

### Saturated acidic / subsurface lake preparation

FeSO_4_ × 7H_2_O (17.5 mM, 4.87 g, Fisher Scientific, Fairlawn, NJ), followed by MgSO_4_ × 5H_2_O (17.5 mM, 2.11 g, Fisher Scientific) and NaCl (600 mM, 26.02 g, Fisher Scientific) were dissolved in 1 liter of UHP argon sparged ddH_2_O, and filtered via a 500 mL Rapid Top Filter with a 0.2 μm aPES membrane (ThermoFisher) for sterility. 10 N H_2_SO_4_ (Fisher Scientific, Fairlawn, NJ), was then used to bring the lake solution pH down to a pH 3.0 (Fisherbrand FE150 pH monitor). The lake fluid (135 mL) was then added to a previously 10% HCl/MeOH sterilized, custom-made, glass jacked reaction vessels and sparged with UHP argon until 5.0% D.O. or less was maintained while being chilled to 1.2–1.7 °C. The remaining acidic lake fluid was stored in a 2 °C fridge until further use. See also Supplementary Table S1 for a summary of physicochemical parameters with citations.

### Saturated / reduced lake effluent preparation

NaS_2_ × 9H_2_O (25 mM, 1.20 g, J.T. Baker, Phillipsburg, NJ), followed by elemental S (25 mM, 0.64 g, Carolina, Charlotte, NC) were dissolved in 200 mL of ddH_2_O. The solution was boiled and stirred on a heat block until a deep brown color (color change from clear to brown to golden yellow to dark brown, indicating long chain polysulfides) was achieved, and the effluent solution was then allowed to cool in the fume hood. Once cooled, the effluent appears a golden yellow color, indicating polysulfides (S_x_). Values for pH were recorded and adjusted if needed to a pH of 10 with 0.1 M HCl. The effluent solution was then sparged with UHP argon (Gilmore Liquid Air, Pomona, CA) until a 3.5% D.O. or less was achieved, before being siphoned/capped into a previously sparged plastic 60 mL syringe (Dynarex, Orangeburg, NY) for later use. Syringe pumps (KDScientific Model 210, Holliston, MA; New Era Syringe Model 300, Farmingdale, NY) were set to dispense at a 1 mL/h rate. The effluent was stored in an autoclaved screwcap container with a UHP argon sparged headspace until further use in the fume hood. Effluent concentrations and pH values were based on alkaline vent effluent studied on Earth, with the goal of forming polysulfides for stronger FeS formation (Edgcomb et al., 2004; White et al., 2015; Lin et al., 2016; Hermis et al., 2021; Dickson et al., 2022).

Akin to ocean fluid undergoing backflow within seafloor mantle, rock-water interactions, and then being ejected as vent effluent, the ice shell lake “effluent” was modeled as subsurface ocean fluid traveling through fractures into the lake bottom. The vertical syringe setup inherently results in some backflow of ocean fluid into the effluent fluid in the syringe, whereby slightly oxidized reservoir fluid mixes at the syringe tip and interact with sulfides from the reducing ocean fluid to form iron oxides and iron sulfides for subsurface lake precipitate. These precipitates then get injected into the subsurface lake fluid as ice fracture effluent, and excess sulfides further interact with other cations (Fe/Mg) in the solution.

### Strain selection and culturing

*Sulfolobus acidocaldarius (Saci)* GG12_C01_15 strains were first grown to log-phase (OD_650_ = 0.15–0.30) before and after introduction to the acidic lake model. The 16S rRNA was amplified using primers 348Fa (5′-TCCAGGCCCTACGGG-3′) and 1100R (5′-AGGGTTGCGCTCGTTG-3′). PCR conditions were as follows: 5 min of initial denaturation at 95 °C, followed by 30 cycles of 30 s denaturation at 95 °C, 60 s annealing at 48 °C, and 60 s of extension at 72 °C followed by a final extension of 10 min at 72 °C. PCR product size was verified on 1% agarose gel before being purified with a QIAquick PCR purification kit (Qiagen, Germantown, MD). The 16S rRNA PCR products were sequenced using primer 515F (5-GTGYCAGCMGCCGCGGTAA-3′). Sequence data was analyzed using the NCBI BLASTn suite to discern a 99% or higher strain identity match.

Prior to lake vent inoculation, *Saci* was first grown in modified Brock's media (Brock et al., 1972) supplemented with tryptone and sucrose to mid-log phase (OD_650_ = 0.150, Supplementary Table S2). Cells were then pelleted, placed in fresh media, and allowed to incubate in 50 mL conicals while in a slow-moving carousel until OD_650_ increased. Cells were incubated in 0 °C conditions for 3 weeks before being re-passaged in fresh Brock's at 0 °C for two additional generations. If the third generation showed OD_650_ evidence of growth, the cells were plated and re-cultured in fresh Brock's media (at 75 °C) to ensure viability. Blanketed cell growth was visible on plates (TNTC) and in liquid culture (OD_650_ = 0.214) after 5 days of incubation at 75 °C. This evolved strain was then stored in 15 mL conicals in 2–4 °C to be used for acidic lake vent experiments. It should be noted that even after 3 months, this evolved strain broth remained viable for all acidic lake vent experiments 1–6. Biotic experiments 1–3 were carried out with 0.1% sucrose supplementation in Europa lake fluid via 10% Full Brock's media addition, while biotic experiments 4–6 were carried out with Brock's media without any sucrose added to the acidic lake/Brock's media. Finally, abiotic experiments 7–8 included 10% Brock's media without sucrose, and no *Saci* inoculation. Trace simple sugar presence vs. absence became a focus of this study to understand the effects of simple sugars on subsequent biofilm composition, Saci viability, and virus propagation in Europa ocean conditions. Similarly, trace amounts of NZ-amine (a simple amino acid mixture) within the Brock's media (SI) was provided in both sugar present and absent conditions to simulate inhabited conditions whereby cells reuse and recycle material produced by an organic rich ocean, feeding the ice shell via brine veins above.

### Mineral precipitate extractions

Conical tubes were sparged and labeled prior to top layer biofilm extraction. Top layers of biofilm were first removed with a 1,000 mL micropipette into 15 mL conicals. Then, a 50 mL serological pipette was used for a singular precipitate extraction. All tubes were filled to the brim, taped with electric tape, and kept in at 2 °C until further analysis.

### Mineral precipitate characterization

Europa model precipitates were extracted under a UHP argon stream and centrifuged at max speed for 5 min. Raman spectra were then collected on an in-house custom, confocal Raman microscope (Menachekanian et al., 2025), employing a Headwall Raman Explorer spectrometer with ~5 cm^−1^ resolution, an Andor CCD with SOLIS software, a 785 nm narrowband laser and sharp long pass filters from Iridian Spectral Technologies, cutting off at ~50 cm^−1^. A 20x Olympus objective focused the excitation and collected the backscatter, and the sample was placed on a silver slide to avoid glass luminescence and enhance Raman spectra were collected with a laser wavelength at 785 nm, 60 scans of 10 sec each, and plotted as scatter intensity vs. wavenumber using the Origin Software. The Raman laser power was set to < 20 mW. Powder XRD (Bruker Venture D8) characterization under an argon stream was conducted to determine the mineral crystalline structures formed under each Europa condition. Powder X-ray diffraction experiments were conducted on a Bruker Venture D8 single crystal X-ray diffractometer with a Photon II detector and Copper (Cu) radiation from a 3.0 Incoatec microfocus source (IμS) and samples were sealed in 0.5 mm capillary tubes prior to collection. Samples were dried for 4 h at presetting 1 via SpeedVac (Thermo Fisher SpeedVac SPD140DD) prior to analysis. pXRD data were plotted in Origin for analysis. The RRUFF database was used as standards for pXRD mineral characterization (RRUFF, 2025).

### Biofilm sequencing

Biofilm extract was plated on Brock's media supplemented with thermostable Phytagel (Sigma-Aldrich, St. Louis, MO) for 5 days before colonies were picked for broth culture amplification. Broth culture was grown to mid-log phase in fresh Brock's media at 75 °C for 2–3 days before undergoing 16S rRNA PCR as described above. The amplified V4 region of the 16S rRNA molecule was then sent for sequencing using primer 515F and compared to several known *Sulfolobus* species, including the inoculant to ensure correlation and non-contamination.

### Putative STIV detection via western blotting and RT-qPCR

To confirm the putative propagation of STIV from *Saci* lysogens in biofilm, a standard western blot and chemiluminescence based RT-qPCR were conducted, for major capsid protein analyses and relative mRNA transcript quantification (respectively). Biofilm (200 μL) was first treated with 10% trichloroacetic acid (Sigma-Alrich, St. Louis, MO) for 30 min at −20 °C, spun at 16,000 × g for 15 min a 4 °C, then treated with ice cold 80% acetone (twice) for 10 min at −20 °C before being spun again at 16,000 × g for 15 min at 4 °C. Pellets were then allowed to air dry on ice for 30 min before being treated with Laemmli SDS-PAGE loading buffer supplemented with 6M Urea and 2.5% 2-mercaptoethanol (Fisher, Fair Lawn, NJ; Supplementary Figures S4, S5). The samples were then heated at 95 °C for 7 min, iced for 10 min, then loaded in a 12% SDS-PAGE gel (BioRad, Hercules, CA). Standard western blot conditions were used. The viral major coat protein (MCP) was detected with rabbit anti-B345 serum and goat anti-rabbit IgG HRP secondary antibody (Bio-Rad). The western blot was resolved via chemiluminescent detection using SuperSignal™ West Femto Maximum Sensitivity Substrate (Thermo Fisher, Pittsburgh, PA) to discern the presence of STIV MCP in biofilm. Additionally, mRNA was extracted from biofilm with a Purelink RNA Mini Kit following manufacturers' instructions (Invitrogen, Carlsbad, CA). Following DNase treatment, 16S PCR amplification and gel electrophoresis (using previously described conditions) were performed to ensure no contaminating DNA was still present that could interfere with RT-qPCR analyses. Once DNA absence was confirmed, RNA concentration was normalized using a NanoDrop Lite Plus Spectrophotometer (Thermo Fisher) prior to cDNA synthesis with SuperScript III First-Strand Synthesis SuperMix (Invitrogen). cDNA product was used for qPCR analysis, using Sso Advanced Universal SYBR Green Supermix (BioRad), STIV_qPCR_B345_3_F (5-GACAGAATCCAGCCTATCCC-3′), and STIV_qPCR_B345_3_R (5-CGCTTGTCCCGTTAAGATTG-3′). qPCR was run on a CFX Connect Real-Time PCR System, and analyses were performed on CFX Manager Software (BioRad). Note that while viral MCP mRNA transcripts and protein detection of viral MCPs are suggestive of active virus propagation, whole virus could not be imaged in this study, due to lack of access to a TEM instrument.

## Results

### Summary

This work demonstrates that aqueous models based on a constrained set of Europa-based subsurface lake conditions can produce a variety of mineral-based FeS/biosulfur/S_8_ precipitates suggestive of *Saci* viability via extensive biofilm formation. Results include physicochemical dynamics between biotic and abiotic systems, mineral characterization, STIV proteomic analysis, and virion transcriptomic analysis. Physicochemical dynamics include initial vs. final lake pH, temperature, D.O., and FeS precipitate dimensions. All conditions were tested in triplicate (N=3) and examined via Raman, western blot analyses, RT-qPCR, and via viability assays.

### Acidic icy lake vent physicochemical condition design

Ice-ocean subsurface lake model experiments were conducted in aqueous systems both abiotically and with integrated *Saci* microbes. The conditions used within the range proposed for Europa's subsurface lakes, with the intention of probing additional parameters within this range in the future. Initial dissolved oxygen (D.O.) saturations of 3.5–5% in this study are derived from the presumption that molecular oxygen from the surface is leaching into the ice shell and subsequently being trapped in subsurface lakes for microaerophilic conditions (Pasek and Greenberg, 2012; Hand and Brown, 2013). The exact starting D.O.s were aimed to begin at the lowest D.O. achievable with our setup. Lake temperatures of 1–5 °C are based on computational models of tidal fracturing kinetics on the ice shells' convective layer (Chivers et al., 2021; Lawrence et al., 2024), with our exact lake temperature of 1–2 °C aimed for the lower end of these modeled predictions. The lake pH of 3 was based on the detection and presumed leaching of oxidized sulfuric acid from the surface of Europa to subsurface lakes, predicted to be in a pH range of 2–6 (Dalton et al., 2003; Zolotov and Shock, 2003; Pasek and Greenberg, 2012). A pH of 3 is also typical of acidic hot spring conditions where *Saci* can be isolated in nature, as well as a standard control in *Saci* laboratory culturing (Brock et al., 1972; Anderson et al., 2017; Fernandes-Martins et al., 2024a,b).

Iron/magnesium/sodium concentrations are based on a presumed ultramafic mantle leaching of magnesium and iron salts (Carr et al., 1998; Zolotov and Shock, 2003, 2004; Pappalardo et al., 2024). Ferrous and magnesium ion concentrations were 17.5 mM as an averaged median starting point, based on previous acidic (pH 5.5–6) early Earth ocean studies. Of previously probed iron concentrations of 10 mM (White et al., 2015), 20 mM (Hermis et al., 2021), 26.5–58 mM (Carman et al., 2024), 50 mM (Dickson et al., 2022), to 25–75 mM (Barge et al., 2015), we took the median of the lower average range (10–25 mM) previously tested in prior ocean world models, since iron/magnesium concentrations on Europa are not well-constrained. The same principle was applied to magnesium solute concentration, as it was detected on the surface of Europa's ice shell, but is not yet constrained. Provided brine veins and ocean salts concentrating in subsurface lakes from surrounding ice melts, solutes were determined by *Galileo* NIMS data and computational models. Models revealing an Fe-Mg-Na-Cl rich ocean and a O_2_-H_2_SO_4_ rich surface (Chyba and Phillips, 2001; Spaun and Head III, 2001; Hand and Chyba, 2007; Brown and Hand, 2013). The subsurface lake salinity of 600 mM (3.5%) was based on detected sodium chloride salts on the surface of Europa, as well as computational models suggesting an ocean salinity comparable to that on Earth, around 3.5% as well (Kivelson et al., 2000; Brown and Hand, 2013; Hand and Carlson, 2015; Trumbo et al., 2019).

To decouple the effects of physicochemical stress or nutrient limitation, trace amounts of D-sucrose was added to the Europa lake media as a minimal chemoorganotrophic energy source for the first three trials, while NZ-amine (0.1% each) was added for all 8 trials (biotic and abiotic). This allowed us to assess whether reduced viability in *Saci* under Europa analog conditions arose from physicochemical constraints or insufficient bioavailable carbon. Since polysaccharides are also a major source of biofilm production in Saci, we probed if the presence of even 0.1% simple sugars would impact the biofilm morphology within the model. Both sucrose and NZ-amine are stable across a wide range of pHs, and easily accessible in most labs, making them a practical experimental control as a minimal energy source. While no macromolecules, like amino acids or sugars, have been detected on Europa, there is potential for simple organic molecules, as seen on asteroids Bennu (Furukawa et al., 2026) and Ryugu (Potiszil et al., 2023).

Europa's ocean effluent is based on hypothesized brine veins saturated with reduced sulfur compounds and iron snow (Sahai et al., 2024) that accumulates over time at Europa's ice-ocean interface. Sulfide concentrations were selected assuming both endogenous (via Europa's FeS core) and exogenous introduction of sulfur. Previous early Earth studies included added sulfide concentrations of 10 mM (White et al., 2015), 20 mM (Hermis et al., 2021), and 50 mM (Barge et al., 2015) in effluent solutions. Our model added 25 mM elemental sulfur to introduce polysulfide and hydrogen sulfide mid-reaction with acidic subsurface lake fluid. While concentrations are likely saturated compared to actual sulfur in Europa's ocean/brine veins (Zolotov and Shock, 2003; Melwani Daswani et al., 2021), this study provides a starting point for probing various FeS metabolic pathway potentials that could be relevant for modeling abundant sulfur on Europa's surface and within subsurface lakes.

Mineral precipitation, D.O., and pH dynamics in this study were compared before and after microbe inoculation to assess abiotic vs. biogenic geochemistry. Ocean fluid fracture injection rates (1 mL/h) were based on previous ocean world models using syringe pumps (Barge et al., 2015; Hermis et al., 2021; Dickson et al., 2022) as ice fracture fluidics are not well-constrained. Alkaline FeOOH/sulfide-rich “effluent” from the subsurface ocean was initially heated to 80 °C to prevent ocean fluid freezing in the syringe needle, which is upper temperature limit for our plastic syringe. Thermal equilibrium calculations reveal a final injection temperature between 2 and 5 °C, which align well with proposed tidal fracture temperatures (Chivers et al., 2021; Lawrence et al., 2024). Finally, 10-day trials were conducted to allow time for *Saci* biofilm growth for sound viability experiments, as well as time for comprehensive physicochemical dynamic studies in said conditions necessary for long-term microbial habitability studies.

A modified Brock's medium made up 10% of the reservoir fluid (Supplementary Table S1) to support microbial growth and function within the proposed acidic lake conditions. Media contained NZ-amine for simple amino acid supplementation, as well as trace elements vital for most microbial growth. A complex Luria-Bertani broth was previously used for reported microbe integrated vent simulations (Dickson et al., 2022) however a modified Brock's media was applied here to match proposed subsurface lake solutes with magnesium, iron, and sulfate (see Table 1). Solute ranges used in this study were based on Early Earth solute concentrations, as well as *Galileo* probe data alluding to a Fe-Mg-${\text{SO}}_{4}^{2-}$ rich Europa ocean (Supplementary Table S1). Modified Brock's broth (Supplementary Table S2) was added to support *Saci* growth during lake vent incubation. Modified Brocks' chemical composition enables an acidic pH = 3 and ${\text{SO}}_{4}^{2-}$/Mg^+2^/Fe^+2/3^ rich conditions, which fall within the range of conditions proposed for Europa (Brown and Hand, 2013; Thompson et al., 2021).

### Model limitations

This model is intended to serve as a starting point for probing the range of conditions that may be present on Europa that could support habitability, but is by no means comprehensive or definitive. Conditions and concentration ranges on Europa are not all known, but this model provides insights toward Europa-like environments for habitability. The high pressure environment expected in Europa's ocean was outside of the experimental scope of this study, however the higher temperature of the effluent solutions (80 °C) provides some increased pressure to simulate possible high pressure ice cracks. Finally, all *Saci* cells used in this study contained lysogenic virus because STIV is encoded in the *Sulfolobus* genome and would require significant gene editing for virus-free experiments. Due to limitations of the lab, viral detection was limited to mRNA transcriptomics and western blot MCP protein identification. Future studies could involve whole virus imaging via TEM to better support active virus propagation, and not just individual non-infectious (mRNA, MCP) viral particle detection.

### Microbial habitability experiments

**Figure 3:**
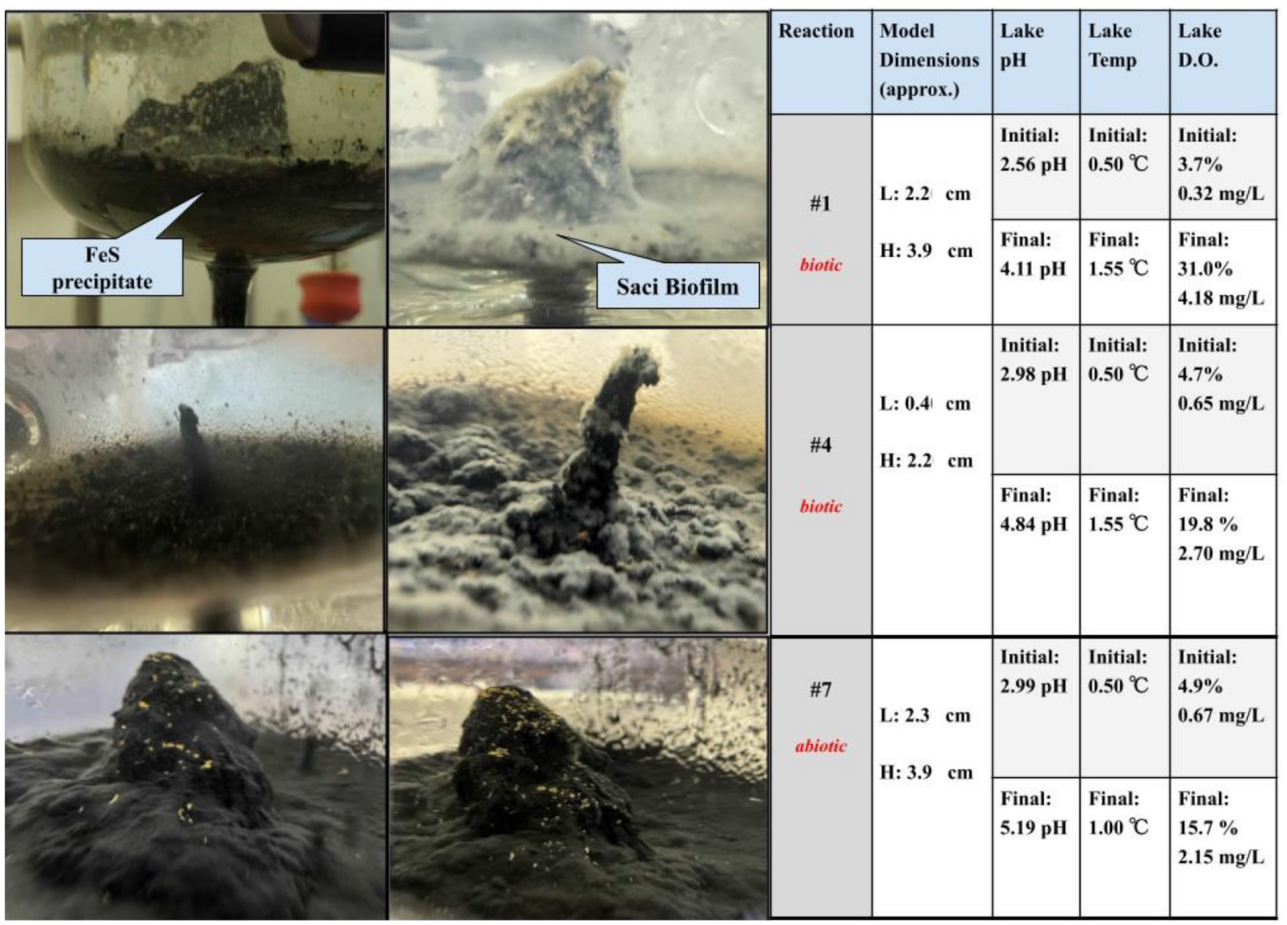
Physicochemical dynamics of Europa subsurface lake fluid model supplemented with 0.1% sucrose **(top)**, without sucrose **(middle)**, and abiotic negative control **(bottom)**. All reactions occurred over 10 days and those with microbes (top & middle) included 5 days without *Saci* inoculation, followed by 5 days of *Saci* integration. Initial parameters were taken at the start of biotic integration (day-1), while final parameters were recorded 10 days postreaction start.

Biofilm growth was observed under all biotic conditions (sugar present and sugar absent) suggesting viability, and precipitation morphologies were unique to each model (Figure 3; Supplementary Figures S1–S3). This suggested that sugar nutrient limitation did not significantly inhibit biofilm growth. Interestingly, final precipitate dimensions varied widely from 2 to 4 cm in height, and final D.O.s deviated from their initial < 5% D.O.s to anywhere from 9.5 to 31%. The increased D.O. over time was likely caused by slow leaks through the Parafilm seal and illuminated striking geochemical differences between FeS oxidated products in biotic vs. abiotic conditions but introduces some uncertainty for defining redox-chemistry and microbial processes. However, such conditions of gradual exposure to molecular oxygen may very well mimic fluid fracture introduction of oxidants from Europa's surface (Pasek and Greenberg, 2012; Brown and Hand, 2013; Hesse et al., 2022). Temperatures stayed relatively constant during the experiments with biotic ranges from 1.50 to 2.00(±0.2) °C and abiotic ranges from 0.80 to 1.00(±0.2) °C.

**Figure 4:**
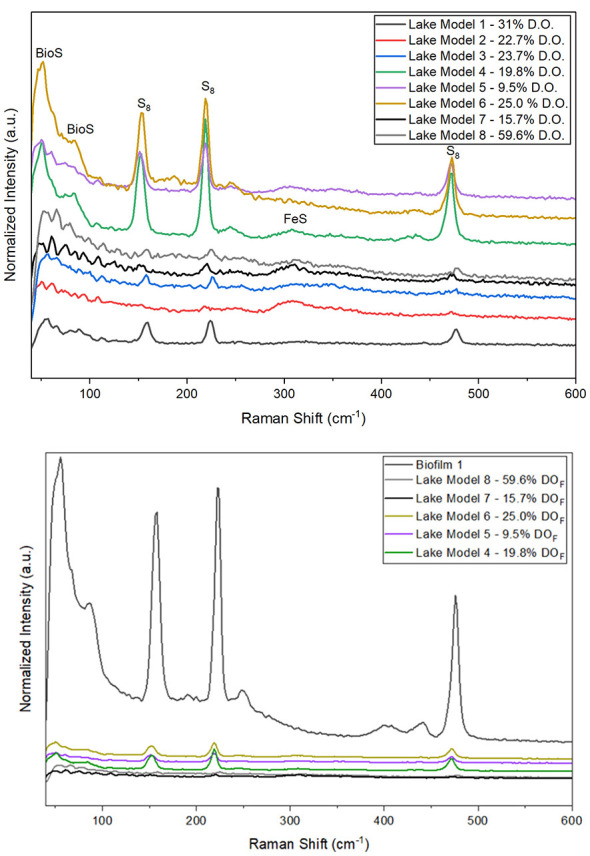
Raman spectra of all 8 wet lake precipitates from each model **(Top)**: sugar present (Models 1–3), sugar absent biosulfur measurements (Models 4–6), abiotic samples (Models 7–8), The lower plot compares pure biofilm biosulfur to sugar absent biofilm, plotted at the same intensity scale. The strong BioS bands below 100 cm^−1^ are a signature of biosulfur, evident in models 4–6 and the biofilm. The wet FeS precipitate was quickly centrifuged and applied to a silver slide before each run.

Unique mineral precipitate morphologies were also observed based on the presence/absence of sugar in *Saci* inoculum (Figure 3). The addition of 0.1% sucrose demonstrated visibly thicker *Saci* biofilm formation in the Europa lake systems (Figure 3; Supplementary Figure S1) compared to sugar absent conditions (Figure 3; Supplementary Figure S2). While in sugar present conditions, films appeared within the first 48 h, sugar absent conditions demonstrated much slower growth, only showing viability after 4–5 days alongside the formation of a light-yellow precipitate. This suggests that sugar nutrient availability affects initial growth rates of microbes. Raman spectroscopy analysis indicated that the light-yellow precipitate was biosulfur (Figure 4), a combination of elemental sulfur (S_8_), and organics, putatively amino acids, adsorbed to polysulfides, which we will label as BioS based on comparisons to published Raman data (Nims et al., 2019; Kuschmierz et al., 2022; Liu et al., 2022). Initial pH values from 2.98 to 2.99 increased to a pH range of 4.11–5.04 by the end of the 10-day trial; however, no significant differences were found between the various abiotic experiments. In microbe-integrated experiments, lake pH values that started in the range of 5.19–5.66 prior to *Saci* inoculation fell to a range of 4.84–5.01 soon after biofilm growth. These results support microbial viability based on biofilm formation in acidic icy lake conditions, and that microbial prevalence can significantly alter the physicochemical dynamics of the subsurface lake it exists within.

### Mineral/geochemical analyses

Mineral precipitates formed within each subsurface lake model were characterized by Raman spectroscopy (Figure 4) and powder X-Ray diffraction (pXRD, Supplementary Figure S6). Vastly different FeS oxidation products emerged in biotic, sugar absent lake models (little yellow powder, Supplementary Figure S2) vs. abiotic, sugar absent lake models (bright orange precipitate, Supplementary Figure S3). While pXRD analysis (Supplementary Figure S6) revealed halite, no other crystallinity was found, hence all elemental sulfur and FeS must be assumed amorphous. While the lake samples have a low concentration of NaCl, the halite is likely the result of the drying process prior pXRD analysis, which was not required for Raman measurements. Raman intensities for sugar absent *Saci* vent systems (lake models 4–6 in Figure 4) reveal sharp low frequency peaks below 100 cm^−1^ which we attribute to biosulfur based on published values (Nims et al., 2019), suggesting organics, putatively amino acids, adsorbed to polysulfides (Nims et al., 2019; Emmert et al., 2024). We also observe prominent standard S_8_ peaks at 153, 219, and 474 cm^−1^ in Figure 4. As expected, abiotic lake models lack sulfur metabolism and hence the 50–100 cm^−1^ BioS bonds are absent, but reveal low intensity peaks at 153, 219, and 474 cm^−1^ indicative of S_8_ and a broad FeS peak at 310 cm^−1^ (Liu et al., 2022; Emmert et al., 2024). Similarly, biotic 0.1% sucrose-supplemented lake models also lack the BioS bonds (50–100 cm^−1^) display low intensity S_8_ (153, 219, and 474 cm^−1^) and an FeS signature around 310 cm^−1^ (MacKenzie et al., 2020; Hand et al., 2022). Even in the presence of sugar, biofilm 1, rich with *Saci* cells containing potential biosulfur globules, has a Raman spectrum with biosulfur signals, notably low frequency wing-tipped BioS bonds, so intense (Figure 4, bottom) that sugar absent precipitate model peaks are barely visible when plotted on the same scale. These results highlight the Raman spectral differences for biosulfur generated from biotic (*Saci* biofilm rich) systems and elemental sulfur from abiotic systems.

### Biofilm virion proteomic analyses

**Figure 5:**
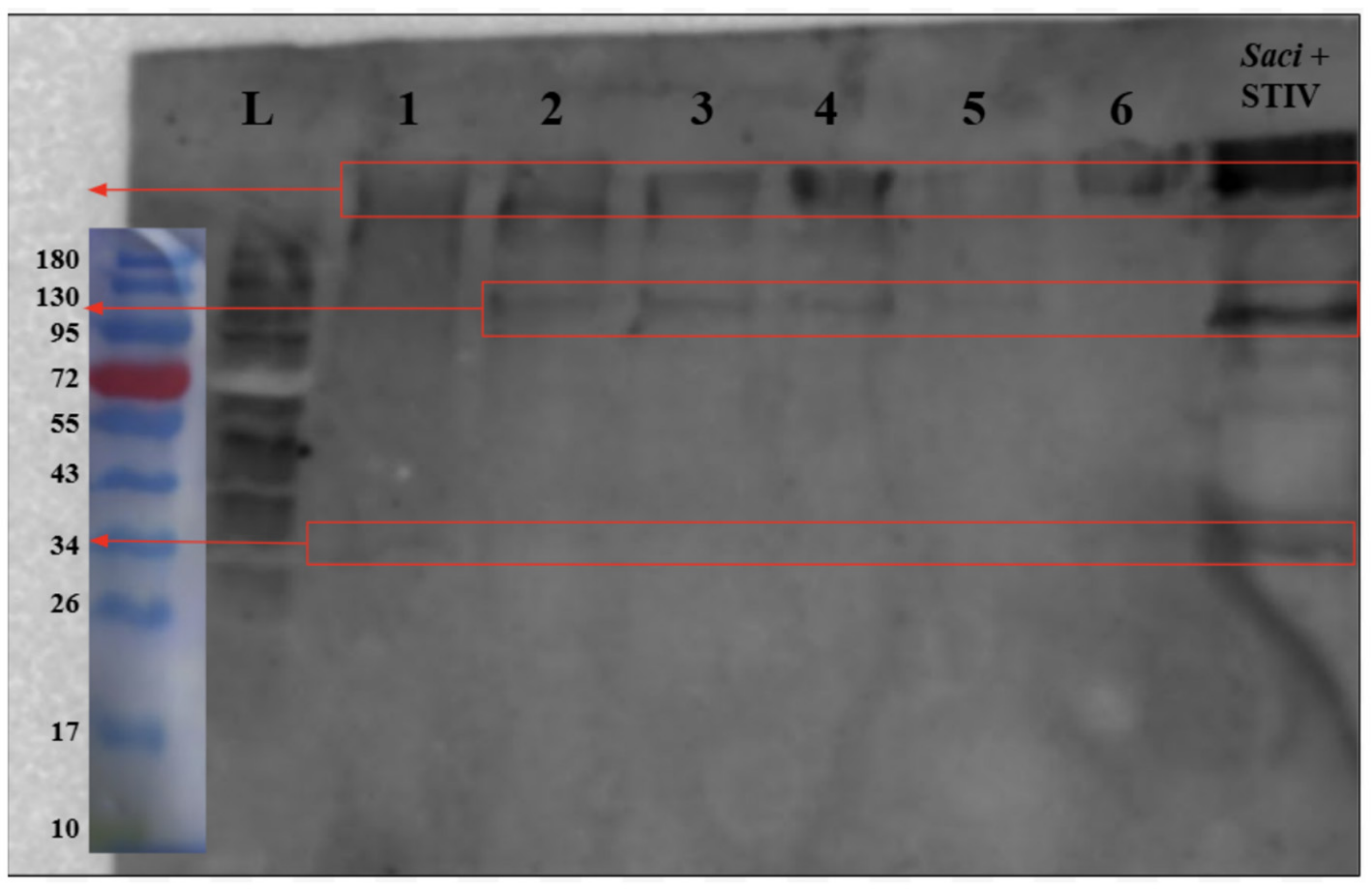
Viral Protein analyses of all six biofilm samples to probe STIV viral particle presence. ECL western blot results of Biofilm samples 1–6 alongside positive control *Saci* with STIV (i.e., Inoculum).

Western blot analyses of proteins extracted and purified from biofilm reveal positive identification of B345 STIV major capsid proteins in all six biofilm samples tested (Figure 5). Chemiluminescent bands at ~37 kDa, 111 kDa, and +200 kDa can be observed in all samples, including the positive control, i.e., previously purified whole STIV particles. All lake aliquots were previously tested from each experiment, but they displayed western results too weak to verify protein presence. This suggests the potential for STIV propagation in all biotic Europa subsurface lake models in this study, and that sugar nutrient limitation was not a key factor specifically when probing biofilm for viral shunt. However, the sugar absent samples resulted in higher average viral mRNA transcript.

### Biofilm virion transcriptomic analyses

**Figure 6:**
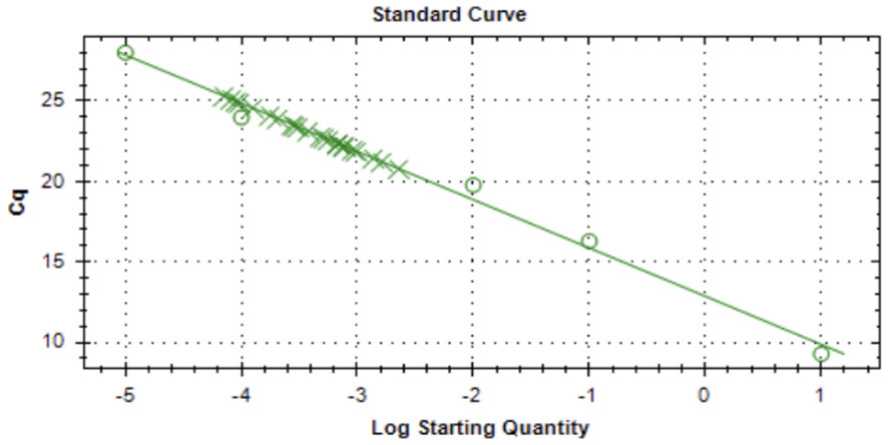
Transcriptomic RT-qPCR results of STIV MCP mRNA detected in the same samples. Figure includes the standard curve (*R*^2^ = 0.990). Table 2 includes average MCP transcripts/mL, based on Cq values.

RT-qPCR analyses reveal positive identification of mRNA transcripts for B345 STIV major capsid transcripts (mRNA) as early as 21 cycles into the qPCR reaction (Figure 6). Virus-free experiments were not conducted because STIV is encoded in the *Sulfolobus* genome and would require significant gene editing to remove. The sample with the highest average STIV mRNA concentration (N=3) was sugar absent Biofilm 4 with ~345,000 mRNAs/mL, while the sample with the lowest concentration of viral mRNA was sugar supplemented biofilm 2 at ~75,000 mRNAs/mL (Table 2). The initial cold tolerant *Saci* inoculum resulted in ~109,000 viral mRNAs/mL at 22.82 Cq, while the negative template control (NTC) revealed a Cq of 35.82, significantly later than the latest sample Cq at 24.06 (Table 2). The *R*^2^ value for this run was 0.99. This is consistent with *Saci* lysogen viability and active STIV propagation under acidic icy lake conditions.

**Table 2:** Average STIV MCP mRNA values from RT-qPCR of biofilm.

Condition	Sample	Avg Cq (*N* = 3)	Avg mRNA/mL (*N* = 3)
Sugar present	Biofilm 1	23.12	81,642
	Biofilm 2	24.06	74,766
	Biofilm 3	22.88	90,336
Sugar absent	Biofilm 4	21.16	344,683
	Biofilm 5	23.32	88,201
	Biofilm 6	22.39	131,600
Positive control	*Saci* + STIV	22.82	109,207
Negative control	NTC	35.82	0

## Discussion

### *Saci* is viable in icy subsurface lake conditions

This work demonstrates that *Saci* is viable in both sugar supplemented and sugar absent subsurface lake conditions that fall within ranges proposed for Europa's ice shell subsurface lake (Figure 3), which establishes a lab-based system for evaluating habitability in icy ocean world environments. Previously, Sulfolobales have individually demonstrated dormancy at 0 °C (Hjort and Bernander, 1999), microaerophilic tolerance at 1.5–24% D.O. (Simon et al., 2009; Fernandes-Martins et al., 2024a,b), and growth at 400 mM NaCl (Ye et al., 2020). However, this study shows not only *Saci* tolerance but also viability at 1–2 °C, 600 mM NaCl, and 9.5–31% D.O. Proteomics and genomics efforts are currently being done to understand exactly how this *Saci* strain can withstand a temperature range of 1–75 °C, a D.O. range of 5 to 31%, and a salinity of 3.5% simultaneously.

From visible *Saci* biofilm formation, TNTC (too numerous to count >300 colonies) viability assays (Supplementary Figures S4, S5), and biosulfur production, it can be suggested that active metabolism of sugars, lipids, proteins, and sulfur by Saci cells likely occurred to facilitate the observation EPS growth. Every biofilm sequencing result was positive for *Saci* with a 99% match (Supplementary Table S3), supporting *Saci* as the biofilm producer. Thicker biofilms were observed for 0.1% sugar supplemented conditions, while thinner biofilms were observed for sugar absent conditions, suggesting that *Saci* metabolized sugar. However, nutrient limitation of sugar was not a key factor in overall microbial viability, as suggested by TNTC viability assays. Given *Saci'*s monosaccharide-rich extracellular polymeric substance (EPS) composition (Kuschmierz et al., 2022), it is possible that even a 0.1% sucrose supplementation supported not only carbohydrate heterotrophy but also thicker biofilm formation (Figure 3). It is also possible that biofilm encapsulated *Saci* were in a state of quiescence, actively metabolizing for EPS and biosulfur synthesis but incapable of undergoing mitosis, until placed in preferred Brock's media at 75 °C. Live cell imaging of biofilm would need to be done to check for actively dividing cells overtime. Nevertheless, the detection of viral mRNA transcripts as well as biosulfur globules and biofilm synthesis demonstrates intracellular activity, demonstrative of cells at the minimum in a state of viability, even though energy or even enough time for active division may not have been provided. In the context of Europa's habitability, while sugar supplemented samples resulted in less intense biosulfur detection, the extensive observed biofilm may support detectable EPS associated biosignatures not explicitly probed in this study.

Prior to this study, no microbe integrated models have included *Saci* and STIV in acidic icy lake or ocean world-like conditions. This study expands on prior microbe (Dickson et al., 2022) and thermally (Hermis et al., 2021) integrated Early Earth studies with another ocean world habitability lens. Our 10-day experiments demonstrate longer putative microbial viability over previous 10-h experiments, which likely would have persisted over extended incubation periods (Dickson et al., 2022). This study determined putative microbial viability via biofilm growth, broth/plate culturing assays, and subsequent 16S sequencing which provides longer-term microbial habitability data as compared to previous viability assays using SYBRGold microscopy hours after microbe-effluent injection (Dickson et al., 2022). This study is unique in combining chilled subsurface lake temperatures, high temperature brine vein effluents, acidic lake conditions with microbes, and conditions inspired by some of the ranges proposed for Europa's ice shell lakes. This work highlights *Saci's* metabolic flexibility and broad tolerance ranges for D.O., temperature, and pH, making it an ideal candidate for continued microbe-integrated ocean world habitability studies.

### Biosulfur is a possible biosignature candidate for habitability in acidic icy lakes and ocean worlds

Raman spectroscopy (Figure 4) revealed strong signals that we attribute to biosulfur in sugar absent precipitate samples and in all biofilm samples, while abiotic amorphous S_8_ and FeS were found in abiotic controls in ranges that were previously identified (Nims et al., 2019). Biosulfur is composed of elemental sulfur (Lima et al., 2013; Nims et al., 2019), polysulfides (Nims et al., 2019), and organo-sulfurs like cysteine and methionine, which so far have not been reported to be abiotically simultaneously generated (Lima et al., 2013; Emmert et al., 2024). So far in nature, biosulfur globules with this low frequency Raman signature have only been reported to be biogenically produced via sulfur metabolizing microbes (Nims et al., 2019; Cron et al., 2021; Liu et al., 2022). In our study, none of the abiotic samples produced Raman signals consistent with the low frequency biosulfur signals, further suggest that these low-frequency signals are of biogenic origin (Figure 4). Consequently, we suggest that biosulfur production could serve as be a candidate biosignature for chemotrophic life on other ocean worlds, and that studies consider low frequency Raman for wet mineral analysis in biotic and abiotic samples for biosignature determination.

While black FeS precipitate was expected to reveal pyrite and mackinawite Raman signals in the 200–300 cm^−1^ range, however broad peaks near the 300 cm^−1^ were revealed, suggesting amorphous FeS formation (Avril et al., 2013). pXRD analysis of the FeS precipitate also showed little diffraction, indicating a high degree of amorphous FeS as opposed to crystalline material (Supplementary Figure S6). Such FeS morphology could be akin to rapidly produced FeS precipitate that has been shown to be more bioavailable to microbial life in acidic hot springs (Luther et al., 2003; Kour et al., 2025), sulfur-rich caves (Macalady et al., 2008), and deep-sea black smoker vent plumes (Dick et al., 2013). Given precipitate from iron snow upwelling, and abiotic sulfuric acid reduction to sulfide, accumulation of nanoparticulate bioavailable FeS precipitate as a nutrient source may prove vital to the habitability of acidic icy ocean-worlds.

### *Saci* metabolism in acidic subsurface lake conditions results in biosulfur accumulation

Lithotrophic metabolism can be supported by the detection of biosulfur, which have so far only been produced biogenically via sulfur-metabolizing microbes (Nims et al., 2019; Liu et al., 2022). So far, no evidence of abiotic biosulfur globule synthesis has been found in nature. Raman spectroscopy of subsurface lake precipitate in this study formed under both abiotic and biotic conditions identified various types of sulfur present. When sugar was added to the medium, high frequency detection of extensive biosulfur in sugar biofilm 1 suggests that at some point in the incubation, sugar/amino acid sources run out, and sulfur oxidation likely becomes the primary reducing agent for the cells (Figure 4, bottom). Sugar absent acidic lake FeS precipitate from this study shows significantly higher intensity Raman signals for biosulfur, with identifying peaks at 80, 260, and 437–441 cm^−1^ (Nims et al., 2019). Such signal suggests the possibility of chemolithotrophy via sulfide oxidation of FeS/HS- to elemental sulfur, as described in previous works (Griesbeck et al., 2002; Duzs et al., 2021). Comparatively, the heavily oxidized abiotic FeS precipitate (Supplementary Figure S3, lake model 8) displays only low intensity Raman signals consistent with S_8_ and FeS and no pronounced biosulfur signatures in the low frequency range (45–100 cm^−1^), suggesting the stunted thermokinetics of HS^−^/S^2−^ → S_8_ under abiotic and low temperature conditions (Figure 4; Supplementary Figure S3). While *Saci* has demonstrated its ability to metabolize S^2−^ → S_8_ → ${\text{SO}}_{4}^{2-}$ under highly aerobic conditions with the help of FAD, quinolones, and sulfide oxidoreductase or SQR (Griesbeck et al., 2002; Fernandes-Martins et al., 2024a,b), it can be hypothesized that under less optimal hypoxic or microaerophilic conditions and in the presence of FeS/HS^−^, acidic lake *Saci* oxidizes S^2−^ → S_8_ which then accumulates over time in the cell cytoplasm as crystalline biosulfur globules (Lima et al., 2013; Nims et al., 2019; Liu et al., 2022). Previous studies have shown that 1.6 g/L concentrations of S_8_ can actually inhibit the growth of *Saci* by impacting the archaea's ability to maintain homeostatic redox states (Fernandes-Martins et al., 2024a). The reduced biofilm density over time in our sulfur encrusted biofilms 4–6 (Supplementary Figure S2) may be a result of similar growth inhibition as compared to sucrose-supplemented films. This also explains why there was less low frequency biosulfur detection in our sugar present samples, as biosulfur did not need to be sequestered or metabolized.

In sugar absent samples and biofilm extracts, high Raman intensities of biosulfur indicate possible sulfur globular nanoparticle diffusion and sequestration inside the cytoplasm of *Saci* cells. This also suggests possible chemolithotrophy by *Saci* biofilm 4–6 in the absence of sucrose, including biofilm 1 once sucrose supplements had been depleted while in 2 °C incubations prior to Raman analyses. In the presence of sugar, sulfur metabolism may not have initially been the primary oxidative metabolism, resulting in weaker to no biosulfur peak recognition in the surrounding FeS precipitate. Surprisingly, however, purified Biofilm 1 (which was sugar supplemented) contained an intense biosulfur signature (Figure 4), dramatically greater than the signal in sugar-absent or abiotic samples. This suggests possible sulfur metabolism after environmental sugar has been depleted, though not to the effect of sulfur secretion within the FeS precipitate, as seen in sugar absent lake models. While *Saci* has shown the ability to reduce iron as a form of chemolithotrophy (Brock and Gustafson, 1976), the FeS precipitate in our model already contained reduced ferrous iron, thereby limiting Saci's ability to further utilize iron as a fuel source. However, heavily reduced sulfur in FeS precipitate was highly bioavailable for Saci, likely making it a primary redox source for ATP production once organics (amino acids and sugars) depleted.

The sulfur analysis of these acidic lake-vent models suggests that *Saci-*like microbes may be chemolithotrophically supported, provided iron sulfide precipitates from iron snow (Sahai et al., 2024) into a shallow, sulfuric acid and D.O. enriched subsurface lake (Brown and Hand, 2013; Hand and Brown, 2013). This parallels Yellowstone's oxygen-poor hot springs where shallow waters and continuous disturbances by rock-water interactions can result in the introduction of ferrous/ferric iron and sulfides, creating iron sulfide minerals for *Sulfolobus* Fe/S oxidation using surface molecular oxygen as the terminal electron acceptor (Fernandes-Martins et al., 2024a). This may support the presence of S_8_ rings lining the outer edges of acidic hot springs in Yellowstone, to where Sulfolobales can also be found further metabolizing elemental sulfur into sulfate (Fernandes-Martins et al., 2024b). While the acidic subsurface lake in this study did not include molecular oxygen introduction via ice radiolysis, leaching of O_2_ during *Saci* inoculation may mimic oxidant cycling within a thin ice shell near Europa's surface, thereby acting as fractured downflow of material for microbial habitability. Such conditions could be occurring on other ocean worlds such as Europa, making subsurface lakes a possible candidate habitable zone for acidophilic microaerophilic microbes capable of FeS sulfur oxidation.

### Putative STIV propagation by *Saci* lysogen occurs even under limited nutrient conditions

From microbe- and virus-integrated subsurface lake experiments, putative STIV propagation may be suggested via both major capsid protein (MCP) detection and mRNA transcriptomic RT-qPCR analyses, with some caveats. Western blot analyses targeting the MCP of the virus revealed positive detection in all biofilm samples (Figure 5). While the expected band size for the MCP is 37.7 kDa (Rice et al., 2004), protein oligomerization was expected given the trimer morphology of the MCP on fully formed virions (Rice et al., 2004; Wirth et al., 2011). As a result, a predicted trimer protein band size of 111 kDa was determined to be evident, both in the biofilm samples and in the positive control (i.e., *Saci* inoculum). While several attempts were made to extract protein and mRNA from lake samples to demonstrate viral shunt, weak western results could not confirm the presence of STIV in lake fluid. Moreover, given the solutes in the acidic lake contents, qPCR could not be performed on lake samples and resulted in flat amplification curves mimicking that of the NTC. For this reason, the biofilm was lysed for viral detection. We presume that *Saci* biofilm successfully trapped STIV in its sticky EPS layer, making it difficult to escape into the lake. While concentrated within the film itself, excessive biosulfur may have also made protein/mRNA extraction difficult to conduct for analyses. This may have resulted in diminished viral titers unrepresentative of STIV's actual influence on *Saci* biofilm morphology. We presume that continuous mixing of planktonic *Saci* and FeS precipitate would have likely inhibited biofilm production, thus suggesting virus propagation in our reservoir system, which will be a future study.

While it is also possible that STIV MCP proteins and active mRNA transcripts could have been produced prior to Europa conditions during cold adaptation passages and preserved in EPS during Europa incubation, *Saci* cells remained viable under refrigeration at mid log phase right up until the point of Europa inoculation. Thus, the possibility of extensive viral shunt during incubation in the fridge is unlikely. Moreover, the OD drops did not occur during 4 °C incubation periods (Rajnovic et al., 2019; Abedon, 2026), which should be expected in the event of viral lysis in planktonic cells. Thus, transcriptomic and proteomic results from this study support the possibility that STIV propagation may have occurred in cells within model Europa lake induced biofilm. However, this conclusion should be viewed putatively and cautiously, given that non-infectious materials, instead of whole virions, were isolated and quantified. Nevertheless, STIV's MCP is composed of a highly conserved double jelly roll morphology, seen not just in other prokaryotic viruses (e.g., PRD1), but also eukaryotic Adenoviruses (Benson et al., 1999; Rice et al., 2001). For this reason, we propose that STIV and its DJR MCP could serve as a model virus for candidate viral biosignature detection methods in future Europa studies.

Using mRNA transcripts, RT-qPCR comparative analyses revealed ~75,000–345,000 viral MCP mRNA transcripts/mL across all six biotic samples (Figure 6). Surprisingly, no direct relationship between the intensity of western protein detection and RT-qPCR mRNA detection could be determined. It is possible that much of the mRNA degraded before analyses were performed for earlier biofilms (samples 1–3). However, the average transcript concentrations between sugar present vs. sugar absent samples differed, where the sugar absent samples had a much higher average of active transcripts extracted from biofilm. The sugar present samples with 0.1% sugar added resulted in visibly increased sticky EPS production. This extensive EPS matrix may have inhibited the spread of viruses across other cells, resulting in a lower average mRNA/mL count. In the sugar absent samples, the biofilm formed was not as robust, perhaps resulting in more efficient cell lysis, attachment, and eventually entry into other cells. In contrast, it is striking that mRNA remained in the biofilm after 3 months of 2 °C biofilm incubation for transcriptomic analyses in the case of Biofilm 1. Continued biofilm viability via EPS degradation for monosaccharide utilization could explain the lasting mRNA constructs prior to cDNA synthesis. Given STIV's conserved MCP double jelly roll morphology in connection with viruses from other (eukaryotic and bacterial) domains of life (Rice et al., 2004), this work suggests that STIV may be a strong model virus for generating possible astro-virological biosignatures in lab-based experiments.

## Conclusion

This work highlights the potential for *Saci* and STIV to serve as a potential microbial model for habitability studies of icy ocean worlds, such as ice shell subsurface lakes on Europa, based on observations of prolonged biofilm growth and putative STIV propagation in an initial set of vent conditions that fall within ranges proposed for Europa's subsurface lakes and related acidic ocean environments. This work suggests the viability of *Sulfolobus acidocaldarius* in a set of acidic (pH 3–5), Fe-Mg-SO_4_ rich, saline (3.5%), FeS rich, and low temperature (1–2 °C) environments over an extended period of 10 days. Extensive biofilm formation, Raman spectroscopy suggesting the formation of biosulfur, likely STIV propagation, and TNTC *Saci* viability assays after model experiments suggest that this set of acidic icy ocean-like conditions could be potentially habitable for certain extreme microbes. This model is not intended to be comprehensive, and some limitations include the ambient pressure, variable D.O., added nutrients, no virus-free control, and uncertainties in system-specific redox dynamics. While STIV mRNA and MCP were not detected in the aqueous lake samples as an example of viral shunt, the positive detection of MCP protein and mRNA transcripts in biofilm samples suggests the possibility for putative STIV propagation within the insulated biofilm niche. However, whole virus imaging would need to be determined in the future for definite STIV viability statements. We propose that STIVs conserved MCP double jelly roll morphology could be a possible biosignature candidate for virus detection on other worlds. Raman analysis differentiated between biosulfur vs. S_8_ detection which may serve as reference data sets for ongoing and future missions that evaluate habitability of other ocean worlds. Infrared, metabolomics, genomics, and proteomics analyses of these acidic lake/biofilm samples and across a wider range of conditions including higher pressures are underway to further explore habitability constraints.

## Data Availability

The 16s rRNA sequencing data presented in the study are deposited in the NCBI repository, accession numbers PZ379628-PZ379634. Additional data are available in the supporting information pdf file and via Zenodo.org at https://doi.org/10.5281/zenodo.17704998.
