# Supplementary material for: Probing the habitability of potential sulfuric acid rich subsurface lakes in Europa's ice shell via Saci/STIV integrated models
Source: Front Microbiol. 2026 May 29;17:1849852. doi: 10.3389/fmicb.2026.1849852 (PMC13260153; doi:10.3389/fmicb.2026.1849852)
Supplement: Supplementary file 1 [file Supplementary_File_1.pdf]

Supporting Information

Probing the Habitability of potential sulfuric acid rich subsurface lakes in Europa's Ice Shell via *Saci/*STIV integrated models

Damara Saggio,[1,2] Christine L. Phung,[2] Moisés Bravo,[2] Timothy C. Corcoran,[2] Jamie C. Snyder,[1] and S. Chantal E. Stieber[2]*
1. Department of Biological Sciences, California State Polytechnic University, Pomona, CA, 91768, USA
2. Department of Chemistry & Biochemistry, California State Polytechnic University, Pomona, CA, 91768, USA

sestieber@cpp.edu

Table of Contents

1. Figures S3

2. Tables S9

1. Figures

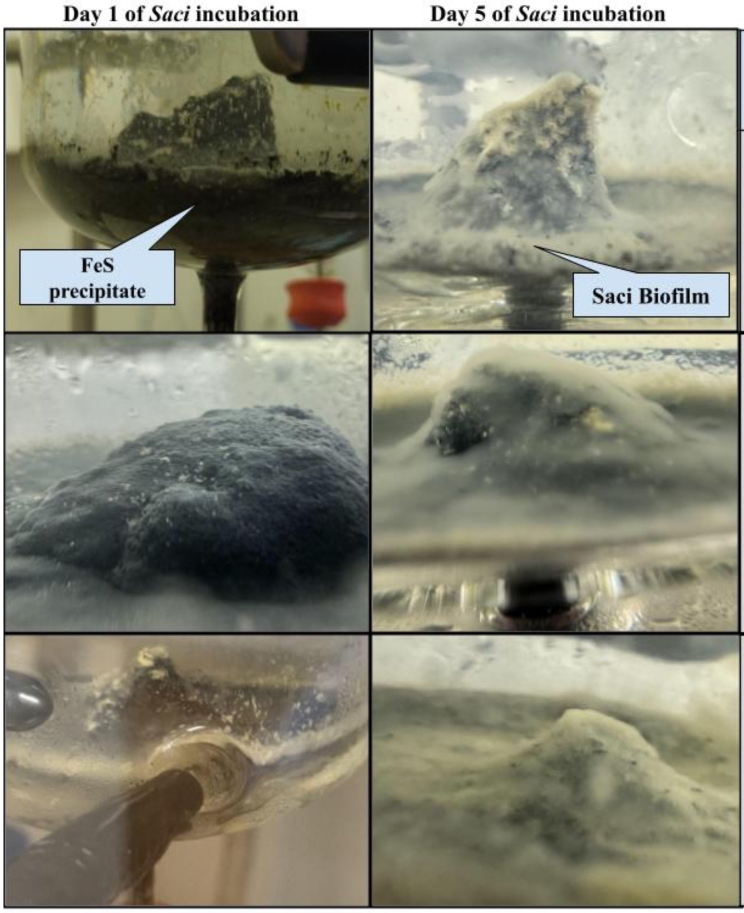

Reaction	Model Dimensions (approx.)	Lake pH	Lake Temp	Lake D.O.
#1	L: 2.2 cm<br>H: 3.9 cm	Initial: 2.56 pH	Initial: 0.50 ℃	Initial: 3.7% 0.32 mg/L
		Final: 4.11 pH	Final: 1.55 ℃	Final: 31.0% 4.18 mg/L
#2	L: 3.5 cm<br>H: 4.0 cm	Initial: 2.56 pH	Initial: 0.50 ℃	Initial: 4.4% 0.60 mg/L
		Final: 5.01 pH	Final: 2.00 ℃	Final: 22.7 % 3.08 mg/L
#3	L: 2.0 cm<br>H: 3.5 cm	Initial: 2.56 pH	Initial: 0.50 ℃	Initial: 4.4% 0.60 mg/L
		Final: 5.04 pH	Final: 2.00 ℃	Final: 23.7 % 3.22 mg/L

Figure S1. Physicochemical dynamics of each Europa subsurface lake fluid supplemented with 0.1% sucrose containing Brock's media. All reactions occurred over 10 days and included 5 days without *Saci* inoculation, followed by 5 days of *Saci* integration. Initial parameters were taken at the start of the reaction (day-0), while final parameters were recorded 10-days post the start of the reaction.

Day 1 of *Saci* incubation **Day 5 of *Saci* incubation**

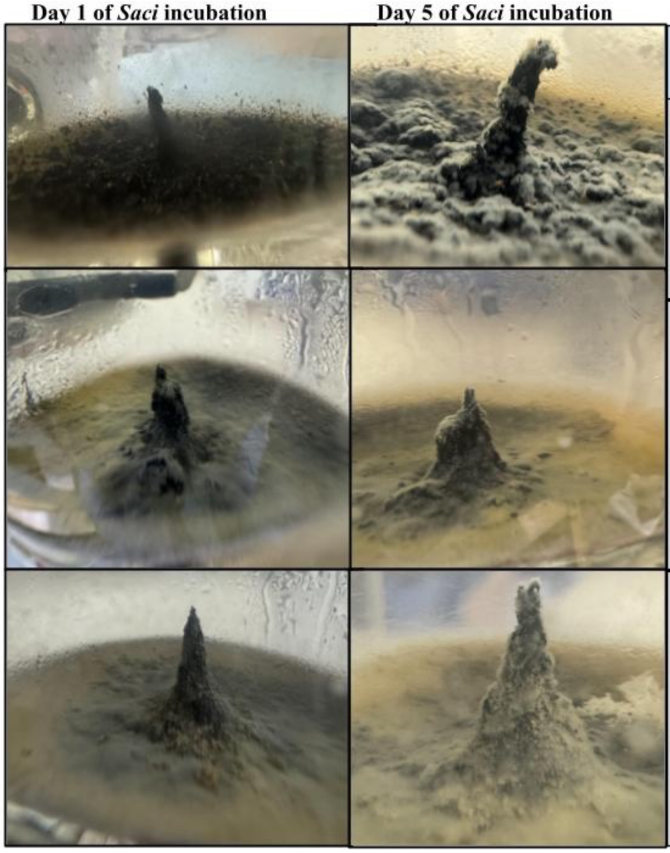

Reaction	Model Dimensions	Lake pH	Lake Temp	Lake D.O.
#4	L: 0.4 cm<br>H: 2.2 cm	Initial: 2.98 pH	Initial: 0.50 ℃	Initial: 4.7% 0.65 mg/L
		Final: 4.84 pH	Final: 1.55 ℃	Final: 19.8 % 2.70 mg/L
#5	L: 1.8 cm<br>H: 4.0 cm	Initial: 2.98 pH	Initial: 0.50 ℃	Initial: 4.7% 0.65 mg/L
		Final: 5 4.95 pH	Final: 1.55 ℃	Final:92.7 9.5% 1.29 mg/L
#6	L: 1.9: cm<br>H: 3.8 cm	Initial: 2.56 pH	Initial: 0.50 ℃	Initial: 4.9% 0.67 mg/L
		Final: 5.01 pH	Final: 1.50 ℃	Final: 25.0% 3.67 mg/L

Figure S2: Physicochemical dynamics of each Europa subsurface lake fluid supplemented with Brock's media without sucrose. All reactions occurred over 10 days and included 5 days without *Saci* inoculation, followed by 5 days of *Saci* integration. Initial parameters were taken at the start of biotic integration (day-1), while final parameters were recorded 10 days postreaction start.

Day 5 of abiotic control **Day 10 of abiotic control**

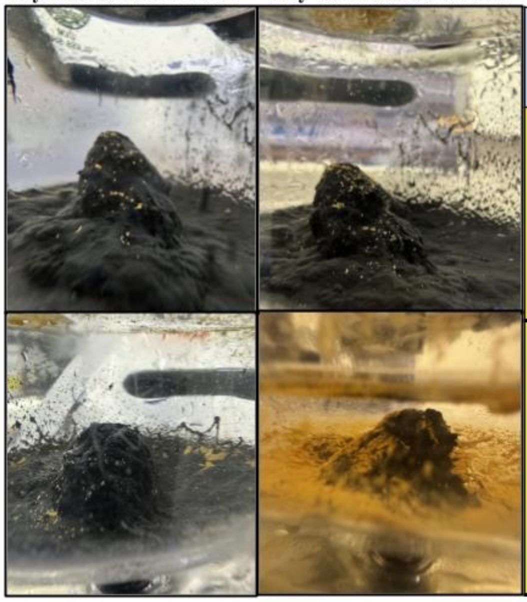

Reaction Number	Model Dimensions (approx.)	Lake pH	Lake Temp	Lake D.O.
#7 *abiotic*	L: 2.3[illegible] cm H: 3.9 cm	Initial: 2.99 pH	Initial: 0.50 ℃	Initial: 4.9% 0.67 mg/L
		Final: 5.19 pH	Final: 1.00 ℃	Final: 15.7 % 2.15 mg/L
#8 *abiotic*	L: 3.2[illegible] cm H: 3.7[illegible] cm	Initial: 2.99 pH	Initial: 0.50 ℃	Initial: 4.9% 0.67 mg/L
		Final: 4.61 pH	Final: 0.80 ℃	Final: 59.6 % 8.15 mg/L

Figure S3. Physicochemical dynamics of Negative Control (abiotic) Europa subsurface lake Vent Models.

Lake Conditions	Europa Trial	Image
Sugar present	1	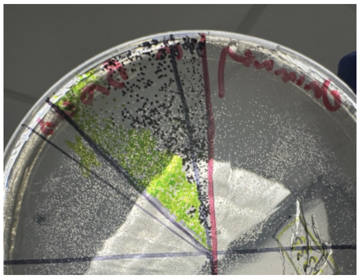 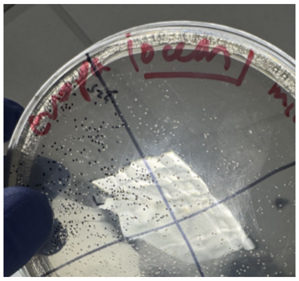 1:1<br>dilution mid-lake sample
	2	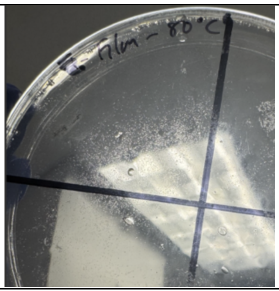 1:1 dilution
	3	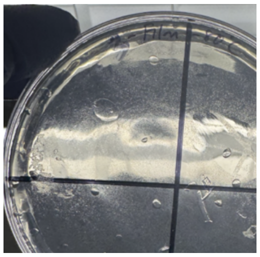 1:100 dilution

Figure S4. Viability assays on Gelrite plates for each Europa lake vent trial where sugar was present.

Conditions	Europa Trial	Image
**Sugar absent**	**4**	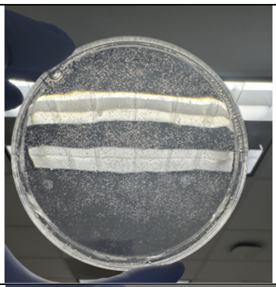 **1:100 dilution**
	**5**	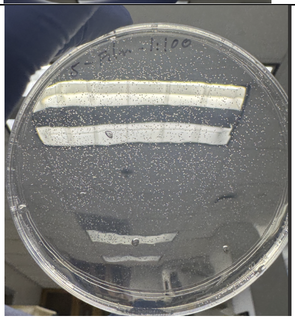 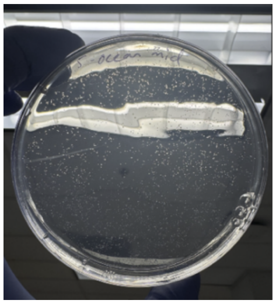<br>**1:100 dilution** **mid lake sample**
	**6**	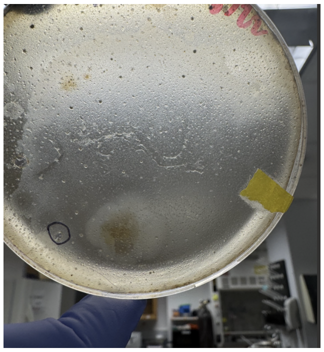<br>**1:100 dilution with STIV plaques extracted for analysis.**

Figure S5. Viability assays on Gelrite plates for each Europa lake vent trial where sugar was absent.

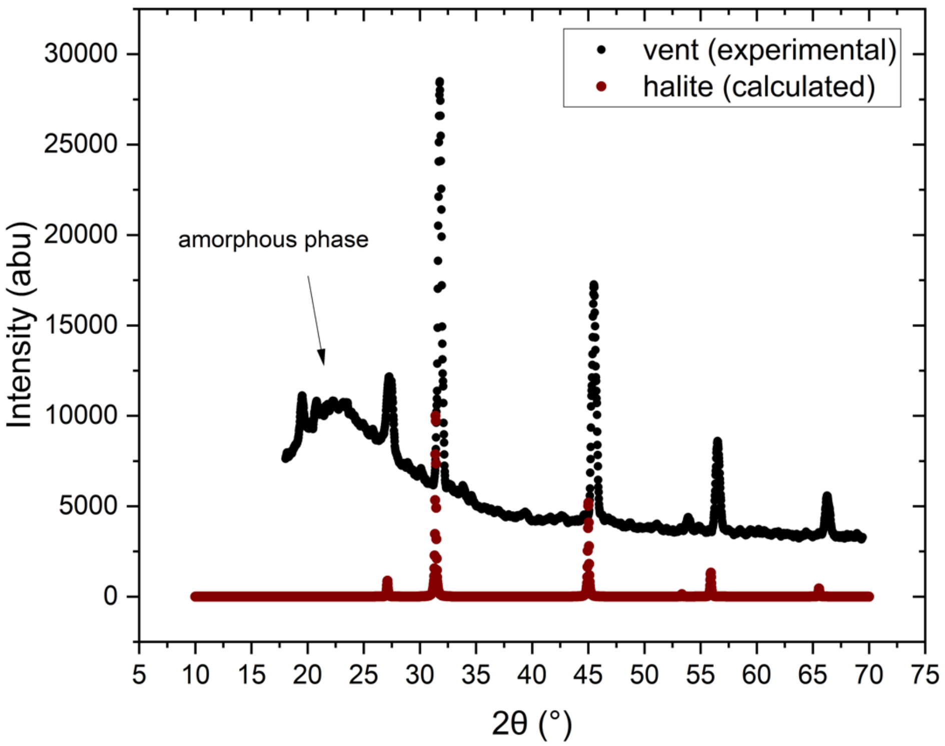

Figure S6. Powder X-ray diffraction data for microbe-integrated precipitate material (black) and calculated halite (dark red).

2. Tables

Table S1: Europa Model Physicochemical Parameters.

Solution	Solute	Quantity
**Europa subsurface lake (135 mL)**<br><br>Initial pH: 3<br>T: 1.5℃<br>DO: <5%	**$FeSO_4$ x $7H_2O$**	**17.5 mM**[4,8,79,106]
	**$MgSO_4$ x $5H_2O$**	**17.5 mM**[8,16,32,79]
	**NaCl**	**3.5%**[32,79]
	**Modified Brock's Media***	**10%**
	**Mid-log *Saci* in Brock's Broth****	**15 mL at OD_{650} = 0.214**
**Saturated lake Effluent (50mL)**<br>pH: 10.5-11<br>T: 80℃ DO: <5%	**Na_2S x $9H_2O$**	**25 mM**[37,80]
	**Elemental S**<br>*Heated to form polysulfides*	**25 mM**[37,80]

*See Table S2 for Modified Brock's Media.
** added via serological pipette after 5 days of mineral precipitation and settlement

Table S2. Modified Brock's media for Europa *Saci* growth.

**Stock solutions**	**Compound**	**Quantity**
**Brock I (x1000)**	$CaCl_2$ x $2H_2O$	70 g/L
**Brock II (x100)**	$(NH_4)_2SO_4$	130 g/L
	$MgSO_4$ x $7H_2O$	25 g/L
	50% H_2SO4	1.5 ml/L
**Brock III (x200)**	KH_2PO_4	56 g/L
	$MnCl_2$ x 4 H_2O	360 mg/L
	Na_2B4O_7 x $10H_2O$	900 mg/L
	$ZnSO_4$ x $7H_2O$	44 mg/L
	$CuCl_2$ x $2H_2O$	12 mg/L
	Na_2MoO_4 x $2H_2O$	6 mg/L
	$VOSO_4$ x 2H2O	6 mg/L
	$CoCl_2$ x $6H_2O$	2 mg/L
	50% H_2SO_4	1.5l g/L
**Iron stock (x100)**	$FeSO_4$ x $7H_2O$	2.8 g/L
**Carbon source**	NZ-Amine	1.0 g/L
**Sugar source (if applicable)**	D-sucrose	1.0g/L

Note: modifications in **red**. Set to pH 3.0 with 10N H_2SO4.

Table S3. Sanger Sequencing results for each Europa lake biofilm sample.*

**Biofilm Sample**	**Sequencing Results (raw, uncut)**
**Saci Inoculum**	NNNNNNNNNNGGTCGGGGGTGATTACTGGGCCTAAAGCGCCTGTAG<br>CCGGCCCACCAAGTCGCCCCTTAAAGTCCCCGGCTCAACCGGGGAA<br>CTGGGGGCGATACTGGTGGGCTAGGGGGCGGGAGAGGCGGGGGGT<br>ACTCCCGGAGTAGGGGCGAAATCCTTAGATACCGGGAGGACCACCA<br>GTGGCGGAAGCGCCCCGCTAGAACGCGCCCGACGGTGAGAGGCGA<br>AAGCCGGGGCAGCAAACGGGATTAGATACCCCGGTAGTCCCGGCTG<br>TAAACGATGCGGGCTAGGTGTCGAGTAGGCTTAGAGCCTACTCGGTG<br>CCGCAGGGAAGCCGTTAAGCCCGCCGCCTGGGGAGTACGGTCGCAA<br>GACTGAAACTTAAAGGAATTGGCGGGGGAGCACCACAAGGGGTGGA<br>ACCTGCGGCTCAATTGGAGTCAACGCCTGGAATCTTACCGGGGGAGA<br>CCGCAGTATGACGGCCAGGCTAACGACCTTGCCTGACTCGCGGAGA<br>GGAGGTGCATGGCCGTCGCCAGCTCGTGTTGTGAAATGTCCGGTTAA<br>GTCCGGCAACGANNNCAACCCNANAANANGNNNNCCGNTTNTTCCCG<br>CGCCTTTTTAGANCGGGGAAAAGCCTCCTTGCGGAGGCACTCGGGGT<br>AGCGCCCTCACGCTTTCGTGCATTGGGGACGTTTCNCNNCTGGTGCG<br>CCNCNNANGNNCTGNAANNATNGGNTTNGTNNATNNTCNNNGNNNNG<br>NNNGNGGNNNNGTTNNNNNNNNNNNNNTGNNN
**Biofilm 1**	NNNNNNGNNNGGTCGGGGGTGATTACTGGGCCTAAAGCGCCTGTAG<br>CCGGCCCACCAAGTCGCCCCTTAAAGTCCCCGGCTCAACCGGGGAA<br>CTGGGGGCGATACTGGTGGGCTAGGGGGCGGGAGAGGCGGGGGGT<br>ACTCCCGGAGTAGGGGCGAAATCCTTAGATACCGGGAGGACCACCA<br>GTGGCGGAAGCGCCCCGCTAGAACGCGCCCGACGGTGAGAGGCGA<br>AAGCCGGGGCAGCAAACGGGATTAGATACCCCGGTAGTCCCGGCTG<br>TAAACGATGCGGGCTAGGTGTCGAGTAGGCTTAGAGCCTACTCGGTG<br>CCGCAGGGAAGCCGTTAAGCCCGCCGCCTGGGGAGTACGGTCGCAA<br>GACTGAAACTTAAAGGAATTGGCGGGGGAGCACCACAAGGGGTGGA<br>ACCTGCGGCTCAATTGGAGTCAACGCCTGGAATCTTACCGGGGGAGA<br>CCGCAGTATGACGGCCAGGCTAACGACCTTGCCTGACTCGCGGAGA<br>GGAGGTGCATGGCCGTCGCCAGCTCGTGTTGTGAAATGTCCGGTTAA<br>GTCCGGCAACGANNNCAACCCNANNACNNGCNCNCCGTTTATTCCCC<br>CGGCTTTTTAGAGCGGGGAAAAGCCTCCTTGCGGAGGCACTCGGGG<br>TAGCGNCCTCACGCTTTCGTGCATTGGNGNCGTTTCCCNNCTGGTGC<br>GCCNNNANGGNCTGNAANGTNNNANNNNGCNNNTNNNNTCNNNNNN<br>NTNNNTCGNNTCNNTTGNNNTTNTCNNGNCNNNNNNCTNNNGNCTTN<br>GNNCTNNNNNNNNNTNNNGNNAGGGCNNGTNGNNNNNNNNNTNNTGT<br>CNNNNNCTTNNNTNANNNTTNTNGTNGNNNNTNNNNGTTNNGGNAGN<br>NNNNNNNTTGNAANTNTNNNNNNNTNNNNNNNNNNNGNN
**Biofilm 2**	NNNNNNNNNNNNATGNCGGGGTGATTACTGGGCCTAAAGCGCCTGT<br>AGCCGGCCCACAAGTCGCCCCTTAAAGTCCCCGGCTCAACCGGGGA<br>ACTGGGGGCGATACTGGTGGGCTAGGGGGCGGGAGAGGCGGGGGG<br>TACTCCCGGAGTAGGGGCGAAATCCTTAGATACCGGGAGGACCACCA<br>GTGGCGGAAGCGCCCCGCTAGAACGCGCCCGACGGTGAGAGGCGA<br>AAGCCGGGGCAGCAAACGGGATTAGATACCCCGGTAGTCCCGGCTG<br>TAAACGATGCGGGCTAGGTGTCGAGTAGGCTTAGAGCCTACTCGGTG<br>CCGCAGGGAAGCCGTTAAGCCCGCCGCCTGGGGAGTACGGTCGCAA<br>GACTGAAACTTAAAGGAATTGGCGGGGGAGCACCACAAGGGGTGGA

	ACCTGCGGCTCAATTGGAGTCAACGCCTGGAATCTTACCGGGGGAGA CCGCAGTATGACGGCCAGGCTAACGACCTTGCCTGACTCGCGGAGA GGAGGTGCATGGCCGTCGCCAGCTCGTGTTGTGAAATGTCCGGTTAA GTCCGGCAACGACNNCAACCCTANNN
**Biofilm 3**	NNNNNNNNNNNNATGTCGGGGTGATTACTGGGCCTAAAGCGCCTGTA GCCGGCCCACCAAGTCGCCCCTTAAAGTCCCCGGCTCAACCGGGGA ACTGGGGGCGATACTGGTGGGCTAGGGGGCGGGAGAGGCGGGGGG TACTCCCGGAGTAGGGGCGAAATCCTTAGATACCGGGAGGACCACCA GTGGCGGAAGCGCCCCGCTAGAACGCGCCCGACGGTGAGAGGCGA AAGCCGGGGCAGCAAACGGGATTAGATACCCCGGTAGTCCCGGCTG TAAACGATGCGGGCTAGGTGTCGAGTAGGCTTAGAGCCTACTCGGTG CCGCAGGGAAGCCGTTAAGCCCGCCGCCTGGGGAGTACGGTCGCAA GACTGAAACTTAAAGGAATTGGCGGGGGAGCACCACAAGGGGTGGA ACCTGCGGCTCAATTGGAGTCAACGCCTGGAATCTTACCGGGGGAGA CCGCAGTATGACGGCCAGGCTAACGACCTTGCCTGACTCGCGGAGA GGAGGTGCATGGCCGTCGCCAGCTCGTGTTGTGAAATGTCCGGTTAA GTCCGGCAACGAGNGCAACCCTANNNNN
**Biofilm 4**	NNNNNNNNNTNATGTCGGGGTGATTACTGGGCCTAAAGCGCCTGTAG CCGGCCCACCAAGTCGCCCCTTAAAGTCCCCGGCTCAACCGGGGAA CTGGGGGCGATACTGGTGGGCTAGGGGGCGGGAGAGGCGGGGGGT ACTCCCGGAGTAGGGGCGAAATCCTTAGATACCGGGAGGACCACCA GTGGCGGAAGCGCCCCGCTAGAACGCGCCCGACGGTGAGAGGCGA AAGCCGGGGCAGCAAACGGGATTAGATACCCCGGTAGTCCCGGCTG TAAACGATGCGGGCTAGGTGTCGAGTAGGCTTAGAGCCTACTCGGTG CCGCAGGGAAGCCGTTAAGCCCGCCGCCTGGGGAGTACGGTCGCAA GACTGAAACTTAAAGGAATTGGCGGGGGAGCACCACAAGGGGTGGA ACCTGCGGCTCAATTGGAGTCAACGCCTGGAATCTTACCGGGGGAGA CCGCAGTATGACGGCCAGGCTAACGACCTTGCCTGACTCGCGGAGA GGAGGTGCATGGCCGTCGCCAGCTCGTGTTGTGAAATGTCCGGTTAA GTCCGGCAACGAGNGCAACCCTANNNNNAN
**Biofilm 5**	NNNNNNNNNNATGNTCGGGGTGATTACTGGGCCTAAAGCGCCTGTAG CCGGCCCACCAAGTCGCCCCTTAAAGTCCCCGGCTCAACCGGGGAA CTGGGGGCGATACTGGTGGGCTAGGGGGCGGGAGAGGCGGGGGGT ACTCCCGGAGTAGGGGCGAAATCCTTAGATACCGGGAGGACCACCA GTGGCGGAAGCGCCCCGCTAGAACGCGCCCGACGGTGAGAGGCGA AAGCCGGGGCAGCAAACGGGATTAGATACCCCGGTAGTCCCGGCTG TAAACGATGCGGGCTAGGTGTCGAGTAGGCTTAGAGCCTACTCGGTG CCGCAGGGAAGCCGTTAAGCCCGCCGCCTGGGGAGTACGGTCGCAA GACTGAAACTTAAAGGAATTGGCGGGGGAGCACCACAAGGGGTGGA ACCTGCGGCTCAATTGGAGTCAACGCCTGGAATCTTACCGGGGGAGA CCGCAGTATGACGGCCAGGCTAACGACCTTGCCTGACTCGCGGAGA GGAGGTGCATGGCCGTCGCCAGCTCGTGTTGTGAAATGTCCGGTTAA GTCCGGCAACGAGCGCAACCCTANAAANANANN
**Biofilm 6**	NNNNNNNNNNNNNNATGNCGGGGTGATTACTGGGCCTAAAGCGCCTG

	TAGCCGGCCCACCAAGTCGCCCCTTAAAGTCCCCGGCTCAACCGGG GAACTGGGGGCGATACTGGTGGGCTAGGGGGCGGGAGAGGCGGGG GGTACTCCCGGAGTAGGGGCGAAATCCTTAGATACCGGGAGGACCA CCAGTGGCGGAAGCGCCCCGCTAGAACGCGCCCGACGGTGAGAGG CGAAAGCCGGGGCAGCAAACGGGATTAGATACCCCGGTAGTCCCGG CTGTAAACGATGCGGGCTAGGTGTCGAGTAGGCTTAGAGCCTACTCG GTGCCGCAGGGAAGCCGTTAAGCCCGCCGCCTGGGGAGTACGGTCG CAAGACTGAAACTTAAAGGAATTGGCGGGGGAGCACCACAAGGGGT GGAACCTGCGGCTCAATTGGAGTCAACGCCTGGAATCTTACCGGGGG AGACCGCAGTATGACGGCCAGGCTAACGACCTTGCCTGACTCGCGG AGAGGAGGTGCATGGCCGTCGCCAGCTCGTGTTGTGAAATGTCCGGT TAAGTCCGGCAACGAGCGCAACCCTANNAANNNNN
**Biofilm 7**	NNNNNNNNNNNNNATGTCGGGGTGATTACTGGGCCTAAAGCGCCTGTA GCCGGCCCACCAAGTCGCCCCTTAAAGTCCCCGGCTCAACCGGGGA ACTGGGGGCGATACTGGTGGGCTAGGGGGCGGGAGAGGCGGGGGG TACTCCCGGAGTAGGGGCGAAATCCTTAGATACCGGGAGGACCACCA GTGGCGGAAGCGCCCCGCTAGAACGCGCCCGACGGTGAGAGGCGA AAGCCGGGGCAGCAAACGGGATTAGATACCCCGGTAGTCCCGGCTG TAAACGATGCGGGCTAGGTGTCGAGTAGGCTTAGAGCCTACTCGGTG CCGCAGGGAAGCCGTTAAGCCCGCCGCCTGGGGAGTACGGTCGCAA GACTGAAACTTAAAGGAATTGGCGGGGGAGCACCACAAGGGGTGGA ACCTGCGGCTCAATTGGAGTCAACGCCTGGAATCTTACCGGGGGAGA CCGCAGTATGACGGCCAGGCTAACGACCTTGCCTGACTCGCGGAGA GGAGGTGCATGGCCGTCGCCAGCTCGTGTTGTGAAATGTCCGGTTAA GTCCGGCAACGAGNGCAACCCTANNNNN

*Note: Biofilm was cultured via gelrite plating, before being proliferated in Brock’s broth to mid log OD_{650} = 0.1-0.2 and undergoing 16S amplification via PCR. Purified PCR products were then sent out for sequencing.
